# Autonomic function in amnestic and non-amnestic mild cognitive impairment: spectral heart rate variability analysis provides evidence for a brain–heart axis

**DOI:** 10.1038/s41598-020-68131-x

**Published:** 2020-07-15

**Authors:** Paola Nicolini, Daniela Mari, Carlo Abbate, Silvia Inglese, Laura Bertagnoli, Emanuele Tomasini, Paolo D. Rossi, Federico Lombardi

**Affiliations:** 1 Cardiovascular Diseases Unit, Fondazione IRCCS Ca’ Granda Ospedale Maggiore Policlinico, Department of Clinical and Community Sciences, University of Milan, Milan, Italy; 2 Geriatric Unit, Fondazione IRCCS Ca’ Granda Ospedale Maggiore Policlinico, Department of Clinical and Community Sciences, University of Milan, Milan, Italy; 3 IRCCS Fondazione Don Carlo Gnocchi, Milan, Italy

**Keywords:** Cognitive neuroscience, Cognitive ageing, Electrophysiology, Electrocardiography - EKG, Autonomic nervous system, Alzheimer's disease, Cerebrovascular disorders, Psychology, Neurology, Physiology, Neurophysiology

## Abstract

Mild cognitive impairment (MCI) is a heterogeneous syndrome with two main clinical subtypes, amnestic (aMCI) and non-amnestic (naMCI). The analysis of heart rate variability (HRV) is a tool to assess autonomic function. Cognitive and autonomic processes are linked via the central autonomic network. Autonomic dysfunction entails several adverse outcomes. However, very few studies have investigated autonomic function in MCI and none have considered MCI subtypes or the relationship of HRV indices with different cognitive domains and structural brain damage. We assessed autonomic function during an active orthostatic challenge in 253 oupatients aged ≥ 65, [*n* = 82 aMCI, *n* = 93 naMCI, *n* = 78 cognitively normal (CN), neuropsychologically tested] with power spectral analysis of HRV. We used visual rating scales to grade cerebrovascular burden and hippocampal/insular atrophy (HA/IA) on neuroimaging. Only aMCI showed a blunted response to orthostasis. Postural changes in normalised low frequency (LF) power and in the LF to high frequency ratio correlated with a memory test (positively) and HA/IA (negatively) in aMCI, and with attention/executive function tests (negatively) and cerebrovascular burden (positively) in naMCI. These results substantiate the view that the ANS is differentially impaired in aMCI and naMCI, consistently with the neuroanatomic substrate of Alzheimer's and small-vessel subcortical ischaemic disease.

## Introduction

Mild cognitive impairment (MCI) is a condition characterised by minor cognitive deficits, without substantial impairment in the activities of daily living, and it represents an intermediate, pre-dementia stage lying along the continuum from normal cognitive ageing to dementia^1^. With the ageing demographic^2^, MCI is set to become a major public health issue.

It has been increasingly recognised that MCI is a heterogeneous syndrome that can be classified into two main clinical subtypes, amnestic MCI (aMCI) and non-amnestic MCI (naMCI), based on whether or not memory is impaired. There is a prevalent agreement, although with some controversy^3^, that they reflect different underlying aetiologies and prognoses, with aMCI regarded as a prodromal form of Alzheimer's disease (AD) dementia and naMCI most likely to progress to non-AD dementias^4,5^. In particular, even if naMCI can evolve to fronto-temporal dementia (FTD) and Lewy body dementia (LBD), there is a growing consensus for a strong association with cerebrovascular disease and vascular dementia (VAD)^5,6^. VAD is the second most common dementia in older people after AD and its subcortical form, due to cerebral small-vessel disease, is the most frequent and clinically homogeneous type of VAD^7^ and the one most likely to be preceded by MCI^8^.

Heart rate variability (HRV) is the physiological phenomenon by which the heart rate changes from beat to beat, producing oscillations in the time intervals between consecutive R waves (RR intervals) on an electrocardiographic (ECG) recording and it reflects the influence on sinus node activity of the two limbs of the autonomic nervous system (ANS), sympathetic and parasympathetic^9^. HRV analysis therefore provides a simple and reliable method for the assessment of autonomic function and has been extensively used in clinical research^10^.

Cognitive and autonomic processes are linked via the central autonomic network (CAN), which is involved both in cognition and in the autonomic regulation of cardiovascular function^11^. The CAN consists of a complex network of cortical and subcortical regions—including the insula, hippocampus and prefrontal cortex—which projects to the preganglionic neurons of the ANS. Thus, the CAN has been widely identified as the neuroanatomic substrate of a brain–heart axis^11,12^.

A relationship between cognitive functioning and HRV has been demonstrated in large cohorts of older individuals^13–15^ as well as in smaller samples of subjects affected by dementia, mainly AD dementia^16^. Despite the potential clinical relevance of autonomic dysfunction in MCI—in terms of syncope/falls^17^, cognitive decline^15^ and mortality^10^—very few studies have evaluated HRV in MCI^18–21^, and none of these have categorised MCI in aMCI and naMCI.

In the current study we hypothesised that the autonomic response to active standing would differ in the two main subtypes of MCI relative to cognitively normal subjects, being attenuated in aMCI and amplified in naMCI. Our hypothesis drew on diverse research on the neural correlates of cognitive domains, on the neuropathological aspects of AD and subcortical ischaemic disease, as well as on the function of specific structures of the CAN, as outlined in the section below.

aMCI is defined by episodic memory impairment, which is the hallmark feature of AD and is associated with damage to the hippocampus^22^, one of the first brain regions affected by neuropathological changes, along with the insula^23,24^. In subjects with aMCI and AD dementia, atrophy and disrupted functional connectivity of both hippocampus and insula have been consistently documented by neuroimaging studies^25–27^ and have been found to be related to memory^27^.

The insula is a key hub within the CAN and a a large body of evidence from animal and human studies corroborates its role in the generation of sympathetic outflow. In rodent models, electrical and chemical stimulation of the insula evoked increases in heart rate (HR) and blood pressure (BP)^28^. In humans, functional neuroimaging has shown that, at rest, insular activity negatively correlated with parasympathetic HRV^29,30^ and that, during tasks eliciting sympathoexcitation, there occurred an activation of the insula which was positively associated with muscle sympathetic nerve activity (MSNA)^31,32^. Likewise, on structural neuroimaging, grey matter volume in the insula negatively correlated with parasympathetic HRV^33^.

The hippocampus has received less attention as a component of the CAN and, even if retroviral tracing techniques have established its connections to the sympathetic system^34^, study findings are sparse. However, despite some amount of discordance (e.g.^32,35^), there appears to be growing support for a contribution of the hippocampus to sympathetic activation . In animals, chemically-induced epileptiform discharges in the hippocampus increased HR and BP^36^ and cholinergic stimulation of the hippocampus increased HRV indices related to sympathetic activation/parasympathetic withdrawal^37^. Also, the stress response in rodents was characterised by an increase in hippocampal electroencephalographic activity which paralleled an increase in HRV indices related to sympathetic activation/parasympathetic withdrawal^38^, and it was reduced by inhibiting hippocampal glutamatergic transmission^39^. In humans, functional neuroimaging studies have demonstrated activation of the hippocampus during sympathetic challenges^31^ as well as negative correlations of parasympathetic HRV with hippocampal activity^30^ and with the grey matter volume of the parahippocampal gyrus^33^. A recent neuroimaging investigation^40^ has reported a negative correlation between the activity in the hippocampus and an HRV complexity index which decreases with sympathetic activation^41^.

In line with these findings, a blunted response to tilt-testing has been reported in subjects with AD dementia^42^ and MCI/AD dementia^21^.

We therefore hypothesised that the autonomic response to active standing would be attenuated in aMCI.

naMCI is typified by impairment in non-memory domains such as attention, executive functioning, visuospatial skills and language. Attention and executive functioning are well known to be dependent on the integrity of prefrontal-subcortical circuits^43^ that course through the white matter and are particularly vulnerable to subcortical ischaemic vascular disease^7^. A wealth of data from human lesion and neuroimaging studies implicate the prefrontal cortex in attention and executive tasks^44,45^. Neuroimaging has also revealed atrophy and reduced connectivity of the prefrontal cortex in subcortical vascular cognitive impairment^46–48^, as well as frontal hypoperfusion in dysexecutive MCI^49^. White matter damage has been found to predict attention and executive dysfunction in subjects with cerebral small vessel disease^50^, vascular MCI^51,52^ and naMCI^53^.

A substantial number of works have converged towards the notion of a neurovisceral integration model in which the activity of the prefrontal cortex can be taken to to index parasympathetic function^11^. In animals, eletrical and chemical stimulation of the prefrontal cortex produced a depressor cardiovascular response^54,55^. In humans, resting parasympathetic HRV has been reported to positively correlate with prefrontal functional connectivity^56,57^ and with electroencephalographic activation of the prefrontal cortex^58^. Parasympathetic HRV has been found to covary with task-related changes in prefrontal cerebral blood flow^56^ and to increase with prefrontal transcranial direct stimulation^59^. During sympathoexcitatory manoeuvres, a deactivation of the prefrontal cortex has been described which positively correlated with MSNA^31^. Higher parasympathetic HRV has been associated with greater cortical thickness in prefrontal regions^56^ as well as better performance on tests of attention and executive functioning^60–64^.

Although there is very scant literature addressing the relationship between ischaemic brain damage and HRV, it mainly points to a parasympathetic dysfunction with sympathetic predominance. In fact, reduced parasympathetic indices and increased indices of sympathetic activation/parasympathetic withdrawal have been found among diabetics with VAD compared to only-diabetic controls^64^ and in subjects with obstructive sleep apnea and white matter lesions (WML)^65^ relative to those without WML . Decreased parasympathetic indices have been reported in the early stages of Binswanger's encephalopathy^66^, in MCI subjects with WML^67^ and in diabetics with lacunar lesions^68^. Indices of sympathetic activation/parasympathetic withdrawal have been shown to be higher in healthy subjects with lower cerebral perfusion^69^.

Since the vagus acts as a "brake" to sympathetic activation^11^, we supposed that a parasympathetic deficit would lead to unrestrained sympathetic activity during an orthostatic stress. We therefore hypothesised that in naMCI subjects there would be an amplified autonomic response to active standing. This would not be at odds with findings of a blunted HRV response to tilt in conditions with sympathetic hyperactivity, such as symptomatic chronic heart failure and unmedicated hypertension^70^ . In fact, an enhanced sympathetic activity already at baseline may exhaust the cardiac sympathetic reserve, i.e. it may limit the ability to further increase sympathetic activity due to a ceiling effect. However, based on our^20^ and others'^18,19,21^ results, we were not expecting baseline differences in HRV indices in MCI subjects, in whom cognitive impairment is slight and dysautonomia is thus likely to be subtle. Also, in hyperadrenergic autonomic disorders like orthostatic hypertension and postural orthostatic tachycardia syndrome, an increased HRV response to standing has been described^71,72^, even in the case of sympathetic predominance at rest^71^.

In this study, which builds on and extends the data of a previous study^20^, we aimed to investigate autonomic function by means of power spectral analysis (PSA) of HRV at rest and during an active orthostatic challenge in the two main clinical subtypes of MCI: aMCI and naMCI. Cognitively normal (CN) subjects were taken as controls. We also sought to explore the relationship of HRV indices with specific cognitive domains, evaluated by neuropsychological testing, as well as with structural brain changes, assessed by validated semiquantitative visual rating scales.

## Results

Table 1 shows the clinical characteristics of the three groups. As expected, scores on the Mini Mental State Examination (MMSE), Montreal Cognitive Assessment (MoCA) and Instrumental Activities of Daily Living (IADL) were significantly lower in MCI subjects than in controls. The prevalence of hypertension and of use of angiotensin-converting enzyme inhibitors/angiotensin receptor blockers (ACE-I/ARB) was significantly higher in the naMCI group. Physical activity was significantly lower in the aMCI group. Trait anxiety, measured by the State-Trait Personality Inventory-trait anxiety (STPI-T) scale, was significantly higher in the naMCI group although there was no difference between groups in the Visual Analogue Scale (VAS) stress score. All subjects had normal blood tests for thyroid function, vitamin B12 and folic acid. None had ischaemic ECG changes, pathological ecocardiographic findings (except mild valvular disease) or haemodynamically significant (> 70%) carotid stenosis.

**Table 1:** Clinical characteristics of the study groups.

	**CN (n = 78)**	**aMCI (n = 82)**	**naMCI (n = 93)**	***P-value***
Age (years)	78.3 (4.8)	79.5 (5.2)	78.9 (5.5)	0.388
Gender, female	63 (80.8)	56 (68.3)	63 (67.7)	0.113
Education (years)	11.5 (4.4)	10.0 (4.1)	10.3 (4.9)	0.078
BMI (kg/m^2^)	24.2 (2.9)	25.3 (4.0)	25.2 (3.9)	0.191
Hypertension	41 (52.6)	57 (69.5)	68 (73.1)	0.013^c^*
SBP (mmHg)	133.9 (16.1)	132.8 (14.7)	131.4 (15.2)	0.749
DBP (mmHg)	75.8 (8.2)	75.0 (7.8)	75.7 (8.0)	0.756
Heart rate, baseline (beats/min)	65.1 (9.0)	65.5 (10.3)	67.4 (9.8)	0.264
Respiratory rate, baseline (cycles/min)	14.5 (2.6)	15.3 (3.2)	15.2 (2.4)	0.136
Respiratory rate, standing (cycles/min)	15.6 (3.0)	16.1 (3.1)	16.5 (2.8)	0.079
Smoking	7 (9.0)	6 (7.3)	6 (6.5)	0.821
Alcohol (AU/day)	1.3 (1.5)	1.0 (1.4)	1.2 (1.4)	0.230
Coffee (cups/day)	1.6 (1.2)	1.2 (1.1)	1.5 (1.1)	0.078
Physical activity (MET-hrs/week)	68.2 (36.9)	52.4 (35.7)	74.1 (45.5)	0.001^a^**^,b^*
Glucose (mg/dl)	90.8 (12.1)	91.7 (11.8)	93.3 (10.2)	0.212
Total cholesterol (mg/dl)	219.2 (37.3)	215.2 (39.8)	220.1 (34.6)	0.657
LDL cholesterol (mg/dl)	133.6 (30.9)	131.6 (35.0)	132.1 (27.8)	0.917
HDL cholesterol (mg/dl)	67.3 (19.1)	62.4 (16.0)	64.6 (17.4)	0.258
Triglycerides (mg/dl)	107.8 (43.0)	110.1 (42.7)	107.7 (43.7)	0.863
FCVDRP score	15.8 (3.2)	16.4 (3.3)	16.3 (3.2)	0.405
FSRP score	13.7 (4.2)	14.3 (3.7)	14.5 (4.6)	0.440
Number of medications	3.4 (1.8)	4.3 (2.6)	4.6 (2.6)	0.012^c^*
Antihypertensive medications				
ACE-I/ARB	31 (39.7)	46 (56.1)	58 (62.4)	0.011^c^**
CCB (dihydropyridines)	9 (11.5)	16 (19.5)	19 (20.4)	0.257
Diuretics	14 (17.9)	23 (28.0)	19 (20.4)	0.271
Psychotropic medications				
SSRI	15 (19.2)	19 (23.2)	30 (32.3)	0.129
Benzodiazepines	13 (16.7)	12 (14.6)	22 (23.7)	0.270
BADL score	5.4 (0.5)	5.5 (0.6)	5.4 (0.5)	0.059
IADL score	7.4 (1.2)	6.3 (1.5)	6.7 (1.4)	< 0.001^b^***^,c^***
MMSE score	28.6 (1.0)	25.8 (2.2)	27.5 (1.9)	< 0.001^a^***^,b^***^,c^***
MoCA score	25.6 (1.7)	21.1 (2.9)	22.9 (2.0)	< 0.001^a^**^,b^***^,c^***
CIRS-m score	2.2 (1.2)	2.1 (1.1)	2.1(1.2)	0.446
STPI-T score	18.5 (5.7)	18.1 (5.0)	20.2 (5.5)	0.016^a^*
GDS-s score	3.3 (3.0)	3.2 (2.8)	3.8 (2.8)	0.179
VAS-stress score	32.5 (22.1)	26.0 (22.1)	29.7 (23.1)	0.187

Continuous variables expressed as mean (standard deviation), categorical variables expressed as n (%). ANOVA or Kruskall-Wallis test for continuous variables. Chi-squared or Fisher's exact test for categorical variables. Pairwise comparisons with Bonferroni corrections.

CN, cognitively normal (controls); aMCI, amnestic mild cognitive impairment; naMCI, non-amnestic mild cognitive impairment; BMI, body mass index; SBP, systolic blood pressure; DBP, diastolic blood pressure; AU, alcohol units (1 AU = 10 g of alcohol); MET, metabolic equivalent (energy expenditure index, 1 MET = 1 kcal∙kg^−1^∙h^−1^); LDL, low density lipoprotein; HDL, high density lipoprotein; FCVDRP, Framingham cardiovascular disease risk profile (score range − 6 to 38, higher scores indicate higher risk); FSRP, Framingham stroke risk profile (score range 0–48 , higher scores indicate higher risk); ACE-I, angiotensin converting enzyme inhibitors; ARB, angiotensin II receptor blockers; CCB, calcium channel blockers; SSRI, selective serotonin reuptake inhibitors; BADL, basic activities of daily living (score range 0–6, higher scores indicate greater functional independence); IADL, instrumental activities of daily living (score range 0–8, higher scores indicate greater functional independence); MMSE, mini mental state examination (score range 0–30, higher scores indicate better cognitive function); MoCA, Montreal cognitive assessment (score range 0–30, higher scores indicate better cognitive function); CIRS-m, cumulative illness rating scale morbidity (score range 0–13, higher scores indicate more severe comorbidity); STPI-T, state trait personality inventory-trait anxiety subscale (score range 10–40, higher scores indicate greater trait anxiety); GDS-s, geriatric depression scale short form (score range 0–15, higher scores indicate greater depressive symptoms); VAS, visual analogue scale (score range 0–100, higher scores indicate greater stress).

^a^Significant difference between aMCI and naMCI, ^b^Significant difference between aMCI and CN, ^c^Significant difference between naMCI and CN, ***P ≤ 0.001, **P < 0.01, *P < 0.05.

Table 2 reports the peripheral vascular burden and the Apolipoprotein E (ApoE) genotypes across the three groups. Although carotid intima-media thickness (IMT) and the prevalence of left ventricular hypertrophy (LVH) just failed to attain statistical significance, left ventricular mass index (LVMI) and percent plaque were, as expected, significantly higher in the naMCI group. ApoE genotyping was available for 55 controls (71% of the CN sample) and for all MCI subjects. As expected, the prevalence of carriers of the E4 allele was significantly higher in the aMCI group (39%) than in either the naMCI (19%) or CN (15%) group.

**Table 2:** Peripheral vascular burden and ApoE genotypes in the study groups.

	**CN (n = 78)**^**†**^	**aMCI (n = 82)**	**naMCI (n = 93)**	***P-value***
Echocardiography				
LVMI (g/m^2^)	83.3 (15.7)	89.3 (20.4)	97.6 (32.8)	0.002^c^***
LVH %	15 (19.2)	19 (23.2)	32 (34.4)	0.061
Carotid ultasound				
IMT (mm)	1.0 (0.1)	1.0 (0.2)	1.1 (0.2)	0.064
IMT > 0.9 mm	68 (87.2)	66 (80.5)	82 (88.2)	0.308
Plaque (%)	22.2 (14.6)	21.0 (15.1)	29.6 (12.6)	< 0.001^a^***^,c^***
ApoE genotype				0.019
E2/E3	5 (9.1)	3 (3.7)	6 (6.5)	
E3/E3	42 (76.4)	47 (57.3)	69 (74.2)	
E3/E4	8 (14.5)	29 (35.4)	17 (18.3)	
E4/E4	0 (0)	3 (3.7)	1 (1.1)	
Carrier	8 (14.5)	32 (39.0)	18 (19.4)	0.001^a^*^,b^**

Continuous variables expressed as mean (standard deviation), categorical variables expressed as n (%). Kruskall-Wallis test for continuous variables. Chi-squared or Fisher's exact test for categorical variables. Pairwise comparisons with Bonferroni corrections.

CN, cognitively normal (controls); aMCI, amnestic mild cognitive impairment; naMCI, non-amnestic mild cognitive impairment; LVMI, left ventricular mass index; LVH, left ventricular hypertrophy; IMT, intima-media thickness; Carrier, carrying at least one E4 allele.

^†^ApoE genotyping available for *n* = 55.

^a^Significant difference between aMCI and naMCI, ^b^Significant difference between aMCI and CN, ^c^Significant difference between naMCI and CN, ***P ≤ 0.001, **P < 0.01, *P < 0.05.

Table 3 shows findings from the assessment of cerebrovascular burden, hippocampal atrophy (HA) and insular atrophy (IA) on neuroimaging. Computed tomography (CT) scans were available for 34 controls (44% of the CN sample) and for all MCI participants except 3 naMCI subjects who refused the exam. Magnetic Resonance Imaging (MRI) scans were available for 14 controls (18% of the CN sample) and for just less than two thirds of the overall MCI subjects (66% aMCI, 60% naMCI). As anticipated, HA was greater in the aMCI group and deep WML (DWML) were more severe in the naMCI group. MRI-based ratings of cerebrovascular burden and HA were consistent with the CT findings (Supplementary Table S1).

**Table 3:** Cerebrovascular burden and hippocampal and insular atrophy on neuroimaging in the study groups.

	**CN (n = 78)**^**¶**^	**aMCI (n = 82)**^**¶¶**^	**naMCI(n = 93)**^**#**^	***P-value***
Fazekas' scale score^†^				
PVWML	1.3 (0.5)	1.7 (0.8)	1.4 (0.6)	0.018^a^*
DWML	1.2 (0.5)	1.4 (0.6)	1.8 (0.7)	< 0.001^a^***^,c^***
Kim's scale score^†^				
Right HA	1.4 (0.8)	2.5 (1.0)	1.4 (0.7)	< 0.001^a^***^,b^***
Left HA	1.7 (0.8)	2.5 (1.0)	1.6 (0.7)	< 0.001^a^***^,b^***
Mean HA	1.6 (0.7)	2.4 (1.0)	1.5 (0.6)	< 0.001^a^***^,b^***
FI rating scale score^‡^				
Right IA	1.6 (0.6)	2.3 (0.6)	1.6 (0.6)	< 0.001^a^***^,b^***
Left IA	1.5 (0.5)	2.1 (0.6)	1.5 (0.4)	< 0.001^a^***^,b^**
Mean IA	1.6 (0.5)	2.3 (0.6)	1.6 (0.5)	< 0.001^a^***^,b^***

Scale scores expressed as mean (standard deviation). Kruskall-Wallis test with Bonferroni-corrected pairwise comparisons.

CN, cognitively normal (controls); aMCI, amnestic mild cognitive impairment; naMCI, non-amnestic mild cognitive impairment; PVWML, periventricular white matter lesions (score range 0–3, higher scores indicate greater WML load); DWML, deep white matter lesions (score range 0–3, higher scores indicate greater WML load); HA, hippocampal atrophy (score range 0–4, higher scores indicate greater HA; FI, frontoinsula; IA, insular atrophy (score range 0–3, higher scores indicate greater atrophy).

^†^On CT.

^‡^On MRI.

^¶^CT available for *n* = 34, MRI for *n* = 14.

^¶¶^CT available for *n* = 82, MRI for *n* = 54.

^#^CT available for *n* = 90, MRI for *n* = 56.

^a^Significant difference between aMCI and naMCI, ^b^Significant difference between aMCI and CN, ^c^Significant difference between naMCI and CN, ***P ≤ 0.001, **P < 0.01, *P < 0.05.

The CT and MRI scans showed no other pathological findings (e.g. large-vessel disease, subdural haematoma, hydrocephalus, tumours). None of the participants had clinical evidence of a cerebrovascular event in the time interval between the acquisition of the scan and the autonomic assessment.

The inter- and intra-rater reliability for visual scale ratings on brain neuroimaging were, respectively, excellent (Cohen's weighted kappa (K_w_) 0.83 to 1) and substantial to excellent (K_w_ 0.78 to 0.90)^73^ (Supplementary Table S2), and conformed to the literature^74–76^.

Table 4 shows the results from the PSA of HRV. The main indices considered were the normalised low frequency power (LFn), the low frequency power (LF) to high frequency power (HF) ratio (LF/HF) and HF. There were no significant differences across the three groups in baseline LFn, LF/HF and HF. In aMCI subjects, compared to naMCI and CN subjects, LFn and LF/HF were significantly lower during active standing and HF was significantly higher. Accordingly, Δ LFn and Δ LF/HF were smaller (i.e. less positive) and Δ HF was larger (i.e. less negative), although the latter was no longer significant after adjustment for relevant covariates. The effect sizes for significant differences in HRV indices were medium to large for LFn and LF/HF (Cohen's d and Glass's delta between 0.8 and 2.5)^77^ and medium for standing HF (Cohen's d = 0.6) (Supplementary Table S3).

**Table 4:** Heart rate variability in the study groups.

	**CN (n = 78)**	**aMCI (n = 82)**	**naMCI (n = 93)**	***P-value***^**†**^	***Q***	***P-value***^**‡**^	***Q***
RR interval (ms)							
Baseline	938.6 (126.3)	941.5 (166.4)	909.9 (135.5)	0.272	0.466	0.433	0.539
Standing	875.5 (129.7)	886.8 (151.2)	865.8 (122.2)	0.693	0.751	0.857	0.857
Δ Standing	− 63.1 (74.3)	− 54.7 (102.4)	− 44.1 (72.1)	0.365	0.505	0.135	0.231
LFn (n.u)							
Baseline	60.5 (16.3)	61.9 (17.2)	59.0 (14.5)	0.751	0.751	0.587	0.640
Standing	74.8 (13.0)	62.9 (18.5)	76.0 (13.7)	< 0.001^a^***^,b^***	< 0.001	< 0.001^a^***^,b^***	< 0.001
Δ Standing	14.3 (10.4)	1.0 (11.6)	17.0 (11.2)	< 0.001^a^***^,b^***	< 0.001	< 0.001^a^***^,b^***	< 0.001
LF/HF							
Baseline	2.2 (1.8)	2.5 (2.3)	1.8 (1.2)	0.379	0.505	0.308	0.462
Standing	4.3 (3.3)	2.8 (2.7)	5.0 (4.0)	< 0.001^a^***^,b^***	< 0.001	< 0.001^a^***^,b^***	< 0.001
Δ Standing	2.2 (2.3)	0.3 (2.2)	3.2 (3.3)	< 0.001^a^***^,b^***	< 0.001	< 0.001^a^***^,b^***	< 0.001
HF (ms^2^)							
Baseline	201.3 (326.9)	288.1 (529.6)	187.9 (284.0)	0.667	0.751	0.449	0.539
Standing	87.2 (145.5)	278.0 (575.6)	87.0 (154.9)	< 0.001^a^***^,b^***	< 0.001	< 0.001^a^***^,b^***	< 0.001
Δ Standing	− 114.1 (254.2)	− 10.1 (227.7)	− 100.9 (188.5)	0.002^a^*^,b^**	0.004	0.102	0.204

HRV indices expressed as mean (standard deviation). Statistical analyses performed on log_10_- transformed values except for Δ HF standing (untransformed).

CN, cognitively normal (controls); aMCI, amnestic mild cognitive impairment; naMCI, non-amnestic mild cognitive impairment; n.u, normalised units; LFn, low frequency power (normalised); LF/HF, ratio of low frequency power (LF) to high frequency power (HF); Δ standing, standing HRV index—baseline HRV index.

^†^ANOVA for all HRV indices except Δ HF standing (Kruskall-Wallis test).

^‡^ANCOVA for all HRV indices except Δ HF standing (non parametric regression). All post-hoc pairwise comparisons performed with Bonferroni correction. *Q* indicates *P*-values corrected for multiple testing with the Benjamini–Hochberg procedure with a 5% False Discovery Rate (FDR).

^a^Significant difference between aMCI and naMCI, ^b^Significant difference between aMCI and CN, ***P ≤ 0.001, **P < 0.01, *P < 0.05.

In the naMCI group the response to the orthostatic challenge was statistically equivalent to that of the CN group. There were no significant differences across groups in the RR interval and in Δ RR.

Total power (TP) showed no statistically significant differences across groups, nor did very low frequency power (VLF) and LF (Supplementary Table S4).

Table 5 displays the results of the correlation analyses in the aMCI group. Δ LFn and Δ LF/HF exhibited a significant positive correlation with the prose-delayed recall Z-score and a significant negative correlation with HA and IA. No significant correlations were found for standing HF.

**Table 5:** Correlations of HRV indices with memory tests and brain atrophy in the aMCI group.

	**Mean (s)**	**Δ LFn (n.u)**			**Δ LF/HF**			**HF standing (ms**^**2**^**)**		
		***r***	***P***	***Q***	***r***	***P***	***Q***	***r***	***P***	***Q***
Prose delayed recall (Z-score)	− 2.32 (1.36)	0.298	0.009	0.025	0.296	0.009	0.025	0.052	0.653	0.712
ROCF-delayed recall (Z-score)	− 1.64 (0.61)	0.185	0.109	0.187	0.121	0.297	0.396	0.136	0.241	0.362
Mean HA score^†^	2.4 (1.0)	− 0.331	0.003	0.017	− 0.331	0.003	0.017	0.027	0.816	0.816
Mean IA score^‡^	2.3 (0.6)	− 0.358	0.011	0.025	− 0.326	0.021	0.042	− 0.071	0.625	0.712

Spearman's correlation analysis adjusted for hypertension, physical activity and trait anxiety. Correlations with memory scores also demographically-adjusted. Cognitive Z-scores and visual rating scale scores expressed as mean (standard deviation).

HRV, heart rate variability; aMCI, amnestic mild cognitive impairment; CT, computed tomography; MRI, magnetic resonance imaging; LFn, low frequency power (normalised); n.u, normalised units; LF/HF, ratio of low frequency power (LF) to high frequency power (HF); Δ HRV index, standing HRV index—baseline HRV index; ROCF, Rey-Osterrieth complex figure; HA, hippocampal atrophy; IA, insular atrophy.

*s*, standard deviation; *r*, Spearman's correlation coefficient; *P*, adjusted *P*-value; *Q*, adjusted *P*-value corrected for multiple testing with the Benjamini–Hochberg procedure with a 5% False Discovery Rate (FDR).

†On CT.

^‡^On MRI.

Table 6 displays the results of the correlation analyses in the naMCI group. Δ LFn and Δ LF/HF exhibited a significant negative correlation with the Digit Cancellation test (DCT) and executive functioning Z-scores and a significant positive correlation with DWML burden. No significant correlations were found for standing HF.

**Table 6:** Correlations of HRV indices with attention and executive tests and cerebrovascular burden in the naMCI group.

	**Mean (s)**	**Δ LFn (n.u)**			**Δ LF/HF**			**HF standing (ms**^**2**^**)**		
		***r***	***P***	***Q***	***r***	***P***	***Q***	***r***	***P***	***Q***
Bell Test (Z-score)	− 3.06 (3.95)	− 0.110	0.300	0.400	0.078	0.463	0.505	− 0.069	0.519	0.519
DCT (Z-score)	− 0.28 (0.68)	− 0.274	0.010	0.024	− 0.309	0.004	0.022	0.080	0.460	0.505
Executive functions (Z-score)	− 0.99 (0.54)	− 0.257	0.016	0.032	− 0.339	0.001	0.016	0.161	0.136	0.204
DWML score†	1.8 (0.7)	0.277	0.009	0.024	0.274	0.010	0.024	− 0.168	0.119	0.204

Spearman's correlation analysis adjusted for hypertension, physical activity and trait anxiety. Correlations with DCT and executive scores also demographically-adjusted. Cognitive Z-scores and visual rating scale scores expressed as mean (standard deviation).

naMCI, non-amnestic mild cognitive impairment; LFn, low frequency power (normalised); n.u, normalised units; LF/HF, ratio of low frequency power (LF) to high frequency power (HF); Δ HRV index, standing HRV index—baseline HRV index; DCT, digit cancellation test; DWML, deep white matter lesions.

*s*, standard deviation; *r*, Spearman's correlation coefficient; *P*, adjusted *P*-value; Q, adjusted *P*-value corrected for multiple testing with the Benjamini–Hochberg procedure with a 5% False Discovery Rate (FDR).

^†^On CT.

## Discussion

To the best of our knowledge this is the first study to investigate autonomic function in the two main clinical subtypes of MCI. We hypothesised, based on the neural substrate of cognitive deficits, that the HRV response to an orthostatic challenge would be attenuated in aMCI and amplified in naMCI, and chose LFn, LF/HF and HF as markers of autonomic activity.

Indeed, in the aMCI group we found a blunted response to active standing, in line with what has been described for tilt-testing in MCI/AD dementia^21^ and AD dementia^42^ subjects. Moreover, we found that this response was significantly associated with evidence of functional (i.e. on neuropsychological assessment) and structural (i.e. on neuroimaging ) damage to AD-vulnerable brain regions such as the hippocampus and insula. In fact, Δ HRV indices correlated (positively) with one episodic memory test (prose-delayed recall) as well as (negatively) with visual ratings of atrophy of both the hippocampus (on CT) and insula (on MRI). The lack of correlation with the other episodic memory test (Rey-Osterrieth Complex Figure (ROCF)-delayed recall) appears consistent with research on AD-related cognitive impairment, demonstrating earlier involvement of verbal than visual memory^26,27^. As far as we aware, the specific association between episodic memory and HRV indices has not been previously highlighted in aMCI subjects. Nonetheless, memory recall has been found to correlate with parasympathetic HRV (negatively) in a mixed MCI-AD/MCI-LBD sample^19^, and with HRV indices of sympathetic activation/parasympathetic withdrawal (positively) in an older cohort^13^. Also, a negative relationship has been reported in late life depression^78^ between the quality of episodic memory and TP at rest (which can be considered a mainly parasympathetic index)^9^.

The magnitude of the effect for significant differences in Δ HRV indices between the aMCI group and the combined naMCI/CN group was found to be medium to large (Supplementary Table S3) and was, therefore, greater than reported for most HRV studies on cognitive impairment^16^, in which effect sizes are predominantly small and large effects are mainly confined to dementias with severe dysautonomia like LBD^79^. The finding that, in our study, the effects sizes in aMCI were not small, as is often the case even in full-blown AD^16^, is presumably due to the fact that we used an orthostatic stressor which can enhance the sensitivity of HRV analysis for autonomic dysfunction.

On the contrary, in the naMCI group we were unable to find the amplified response to active standing we had theoretically anticipated: while the postural changes in HRV indices (LFn and LF/HF) were increased relative to controls, they were not significantly so. However, on correlation analyses we found that Δ HRV indices were associated (negatively) with one attention test (DCT) and with a composite measure of executive functioning as well as (positively) with DWML. No such associations were found in CN subjects .The finding that naMCI subjects had the same response to orthostatic stress as CN subjects is likely due to the fact that the exclusion criteria, inherent to a study on the cerebral substrate of HRV (see “Methods”), selected a low/intermediate cardiovascular risk population in which severe brain vascular burden was under-represented, thus reducing our ability to detect a significant effect. In particular, most studies demonstrating autonomic dysfunction in ischaemic brain damage have focused on subjects at high cardiovascular risk like diabetics with^64^ and without^68^ VAD as well as patients with obstructive sleep apnea^65^, or have not excluded individuals with cardiovascular risk factors and diseases^67^. In our naMCI group at low/intermediate cardiovascular risk, a provocative manoeuvre such as paced breathing, which challenges the parasympathetic system^80^, could have directly assessed the parasympathetic reserve and unmasked mild parasympathetic deficits. However, we decided not to use it because of its potential for bias (see “Methods”). Nevertheless, it should be noted that the correlations observed in the naMCI group were in the expected direction, i.e., they argue in favour of the notion of a parasympathetic deficit causing sympathetic hyperactivation. In fact, indices of sympathetic activation/parasympathetic withdrawal were higher in subjects with poorer performance on tests of frontal lobe function and with more extensive subcortical vascular damage. The lack of correlation with one of the two attention tests (Bell Test) is probably a a consequence of the more restricted score range, which decreases the likelihood of identifying a significant association.

In both the aMCI and naMCI groups, significant correlations of Δ HRV indices with cognitive tests and structural brain changes were small in term of effect sizes (r < 0.3)^81^. This result is commensurate with other studies on resting HRV and cognitive tests in more varied samples (e.g.^60–62^). It is conceivable that, in our case, the ability of an orthostatic challenge to highlight stronger associations was offset by greater sample homogeneity.

There were no significant differences across groups in other HRV indices (mean RR interval, TP, LF and VLF) (Supplementary Table S4).

The lack of significant differences in the the RR and Δ RR intervals is worthy of specific comment and could be due to different reasons. First, HRV is a more reliable estimator of autonomic function than HR^82^ and has proved to be more sensitive in detecting subtle dysautonomia across a range of diseases^83–85^. Indeed, there are numerous reports of no difference in the HR response to an orthostatic challenge in subjects with MCI or even AD dementia (e.g.^21,86,87^). Second, in our study the standing HR (and HRV) was determined from an ECG segment between the 5th and 10th minute of active standing. Since the time course of HR reactivity to standing is characterised by a HR peak at around 10 s and a HR nadir at around 30 s with subsequent stabilisation of HR to values higher than baseline^88^, our protocol was not designed to capture the maximum postural Δ RR and could be missing potentially meaningful differences in RR intervals across the three groups. Third, it can be speculated that in aMCI subjects, the finding of a significant difference in Δ HRV coupled with no difference in Δ RR, could be indicating impaired baroreflex function. However, confirmation of such hypothesis would have required continuous BP monitoring (see Limitations).

The clinical, neuropsychological and vascular burden characteristics of the study subjects were consistent with AD neurodegeneration and vascular cognitive impairment in the aMCI and naMCI groups respectively. They are discussed in the Supplementary Discussion.

Our study has some limitations. Although the use of transformed HRV indices (LFn and LF/HF) finds support in the literature (see “Methods”), it has also been questioned (e.g.^89^). In particular, changes in LFn and LF/HF have been mainly ascribed to changes in HF, implying that transformed indices are not markers of sympathetic activity but rather of parasympathetic withdrawal^89^. However, it is generally recognised that orthostatic stress induces a reciprocal pattern of sympathetic/parasympathetic change^90^ and the conceptual framework of the neurovisceral integration model involves autonomic reciprocity^11^. Also, despite the fact that LFn and LF/HF have been somewhat inconsistently associated with MSNA^89^, they have been shown to exhibit strong positive correlations with the normalised^91^ and absolute^92^ LF powers of MSNA variability during tilt. In our study, it would thus appear appropriate to interpret LFn and LF/HF as indices of sympathetic activation/parasympathetic withdrawal. Moreover, even if our findings fit in nicely with the role of CAN components (insula, hippocampus and prefrontal cortex) in autonomic control, as outlined in the Introduction, alternative inferences can be made should one focus only on the notion of parasympathetic withdrawal, albeit during a manoeuvre designed to activate the sympathetic nervous system . For instance, it might be speculated that lesser parasympathetic deactivation in aMCI stems from deficits of the cholinergic system in AD^93^. Likewise, greater parasympathetic deactivation in naMCI could be due to vagal hyperreactivity secondary to ischaemic damage. In fact, because of the exclusion criteria, subjects with naMCI could be at a prior stage of the disease than those with aMCI, and a compensatory response has been described in early MCI^26^. Anyhow, our results would still provide evidence for differential autonomic impairment in the two clinical subtypes of MCI.

Finally, it must be acknowledged that HF, unlike tranformed indices, is not limited by the assumptions of reciprocity and linearity^89,94^. Yet, the only significant difference we found across groups was in standing HF, and standing HF was not significantly correlated with cognitive tests or structural brain damage in the aMCI and naMCI groups. This was probably the case because HF is a highly dispersed variable^95^. It is possible that different results could have been obtained with a PSA software based on the Welch method, since averaging windowed periodograms has been shown to effectively reduce variance^96^. Similarly, even if traditional HRV indices are recommended as the mainstay of HRV analysis, non-linear measures, which are still not implemented in commercial devices like the one we used, might have provided additional information on the ANS^97^ .

The unavailability of non invasive beat-to-beat BP monitoring precluded the assessment of baroreceptor function^98^, thus leaving a promising avenue of research unexplored. In fact, despite substantive evidence of bottom-up modulation of cortical activity by the central nervous branch of the baroreceptor as well as of top-down influences of rostral brain units on the baroreflex loop, studies on baroreflex sensitivity in cognitive impairment are scarce^99^. Also, findings are inconsistent, with both decreased^100^ and unchanged^21,87^ baroreflex sensitivity reported in AD-related MCI and dementia.

The lack of more sophisticated software, operating in conjunction with respiratory signal recording, may have restricted the number of cases suitable for PSA. As recommended, we excluded subjects with a respiratory rate outside the range of the HF band^101^. Although only very few individuals were lost to analysis (n = 1 in the previous and n = 4 in the current study), such limitation could have been overcome by approaches that centre the HF band around the respiratory frequency (when respiratory rate is > 24 breaths/min)^102^ or that decompose the LF band in respiratory and non-respiratory components (when respiratory rate is < 9 breaths/min)^103^.

The two main clinical subtypes of MCI were assumed to have different underlying aetiologies (AD for aMCI and subcortical small-vessel disease for naMCI) based on well established data from the literature (see “Introduction”), but the diagnosis of MCI due to AD was not confirmed by research biomarker criteria like CSF measures and/or amyloid or perfusion/metabolism neuroimaging^1^. It might be argued that aMCI of the multiple-domain type could represent the longitudinal outcome of both AD and vascular brain damage^104^, and that naMCI could be associated with non-vascular aetiologies^1^. However, several issues deserve mention. First and foremost, findings regarding vascular burden and HA on CT scans as well as the genetic risk of AD (see “Results”) lended support to our hypothesis. In particular, the 39% prevalence of ApoE4 carriers in the aMCI group is very much in line with the 40% value reported for AD^105^. Second, it appears reasonable to posit^4,5^ that multiple-domain aMCI is most likely to progress to AD rather than VAD. In fact, AD is more frequent^7^, has a faster rate of decline^106^ and affects memory and other cognitive domains at an early stage^107^, while memory impairment has been shown to develop late in the course of subcortical vascular disease^108^. This would hold particularly true in a sample like ours at low/intermediate cardiovascular risk. Third, we considered older subjects and used psychiatric and neurological disorders as exclusion criteria, so non-AD related neurodegeneration is unlikely to be relevant in the naMCI group. Indeed, LB pathology is accompanied by visual hallucinations and parkinsonism^109^, and prodromal FTD is young-onset and often presents with behavioural (i.e. psychiatric-like) symptoms^110^. Fourth, atypical non-amnestic variants of AD and mixed AD/vascular processes cannot be ruled out, but the former are rare^111^ and there is evidence that the latter are dominated by AD neuropathology^112^. Fifth, there may be several downsides to biomarkers including invasiveness, limited accessibility and the possibility of their being uninformative (i.e. ambiguous or conflicting) in a substantial proportion of MCI subjects^113^.

The main neuroimaging tool in our study was CT, since all MCI subjects were prescribed a brain CT scan as part of the routine diagnostic work-up (*n* = 172) while not all had a brain MRI scan (*n* = 110), so that focusing on MRI would have required us to reduce the study sample by about 40%. Although CT has practical advantages over MRI, which explain its widespread use in clinical settings, MRI is considered the gold standard for detecting structural brain changes, especially those related to small-vessel disease^114^. Still, the two neuroimaging techniques gave similar results across groups (see Supplementary Table S1 for MRI-based ratings of cerebrovascular burden and HA) and correlations of Δ HRV indices with visual scores of HA and DWML were not significantly different on CT and MRI scans (for all, p > 0.7 with Fisher's r to z transformation test, Supplementary Table S5). In addition, even if MRI would have enabled brain volumetry, visual rating scales have several strengths. They are simpler and less time intensive and can be applied to both CT and MRI^115^. They have been validated against volumetric measures, with which they are strongly correlated^76,115,116^, and exhibit substantial to excellent reproducibility, in the literature and in our specific sample (see “Results”). HA visual rating scales, compared to volumetry, have been shown to be better^117,118^ in discriminating CN from MCI and AD dementia subjects, and to be more accurate predictors of memory performance in community-dwelling older people across the cognitive spectrum^117,119^. This is possibly so because visual inspection encompasses not only the hippocampus, but also the surrounding perihippocampal space, which reflects atrophy of other AD-targeted brain regions such as the parahippocampal gyrus^120^.

As to the possible selection bias arising from the fact that neuroimaging, in particular MRI, was unavailable for some of the subjects (see “Results”), as well as from the fact that MoCA screening was preliminary to neuropsychological testing only in the current enrollment (see “Methods”), it has been addressed by subgroup analyses (Supplementary Table S6–S8) and found to be unlikely to hamper the validity of the Results, as reported in the Supplementary Discussion .

The use of exclusion criteria may limit the generalisability of our findings to the overall MCI population. Also, although the enrollment of subjects with incident (i.e. newly diagnosed) MCI avoids survival bias^121^, the cross-sectional design of the study prevents causal inference. Longitudinal studies will therefore be needed to elucidate the direction of the relationship between cognitive and autonomic dysfunction, as well as to confirm the predictive value of autonomic impairment for adverse events like falls/syncope, progression to dementia, and all-cause and cardiovascular mortality.

In conclusion, our findings substantiate the view that the ANS is differentially impaired in the two subtypes of MCI, in keeping with the neuranatomic substrate of AD and small-vessel subcortical ischaemic disease. In the aMCI group there was a blunted autonomic response to standing. Within the naMCI group functional (i.e. on neuropsychological testing) and structural (i.e. on neuroimaging) cerebrovascular damage was associated with an increase in the autonomic response to standing. These results contribute novel insight into autonomic modulation in MCI and hold potential clinical relevance as autonomic assessment could eventually help to identify subjects at higher risk of adverse health outcomes to whom interventions should be targeted.

## Methods

### Study population

In this cross-sectional study we considered for inclusion 819 community-dwelling older subjects (aged ≥ 65) who consecutively attended a first geriatric visit at the Geriatric Outpatient Unit of our hospital, from January 2016 to September 2017. Referrals were made by general practitioners (GPs) for a wide spectrum of age-related health problems. 120 subjects with a known diagnosis of dementia were excluded, while the remaining 699 were assessed for eligibility by applying a number of exclusion criteria (see later). We thus identified 292 eligible subjects who were screened for cognitive impairment by means of the MoCA^122^. The 228 who screened positive (MoCA score < 26) were invited to undergo neuropsychological testing. Of the 223 subjects who agreed to neuropsychological testing, 34 were diagnosed with dementia and were excluded. The remaining 189 subjects, with a diagnosis of MCI or normal cognition (CN), were asked to take part in the autonomic assessment. Of these 6 declined, so 183 subjects underwent the autonomic assessment and recordings from 173 (*n* = 62 aMCI, *n* = 73 naMCI, *n* = 38 CN) were ultimately considered, after 10 were excluded (see HRV analysis).

Our study includes data from the currently enrolled 173 subjects as well as data from 80 subjects (*n* = 40 MCI, *n* = 40 CN) enrolled in a similar, previous study^20^.

All subjects who accepted the autonomic assessment were also given an ad hoc clinical assessment. All MCI subjects were prescribed a brain CT scan and ApoE genotyping as part of the routine diagnostic work-up for cognitive impairment at our Unit. CN subjects were offered ApoE genotyping as part of ongoing research protocols^123^. For all subjects a standard blood panel was requested (including blood count, glycaemia, lipid profile, kidney and thyroid function, vitamin B and folic acid). Brain CT scans were not prescribed to CN subjects and brain MRI scans were prescribed to MCI subjects by the geriatrician on a case-by-case clinical basis. However, neuroimaging data were also evaluated for those CN subjects who had for some reason (Supplementary Table S9) undergone a brain CT or MRI, and for MCI subjects who had undergone an MRI scan.

Brain neuroimaging was considered only if performed in the previous 6 months. The clinical and autonomic assessments were carried out within one month from the neuropsychological assessment. HRV recordings and brain scans were obtained with the same machines (see later) in the two enrollments.

The rationale for the use of the MoCA as a preliminary screening tool only in the current enrollment is discussed in the Supplementary Methods.

The study was conducted in accordance with the Declaration of Helsinki and was approved by the ethics committee of the Fondazione IRCCS Ca’ Granda Ospedale Maggiore Policlinico in Milan, Italy. All participants gave written informed consent to participation in the study.

The study followed the Strengthening the Reporting of Observational Studies in Epidemiology (STROBE) guidelines for cross-sectional studies^124^.

### Exclusion criteria

Briefly, exclusion criteria were clinical conditions and medications with an established and significant effect on HRV. They have been previously detailed and referenced^20^ and are listed in the Supplementary Methods. It may be appropriate to remark here that, given that the aim of the study was to investigate HRV changes due to cerebral disease, non-cerebral conditions influencing HRV (including some cardiovascular risk factors and all cardiac diseases) were among the exclusion criteria; this methodological premise will inevitably select a sample at low/intermediate cardiovascular risk (see “Discussion”). It is also worth noting that, even though stroke is a potential cause of vascular cognitive impairment, we chose to exclude subjects with a history of stroke because their cognitive profile is very heterogeneous, depending on lesion location^7^, and they more often present with overt dementia^8^. We preferred to focus on subcortical vascular disease which, besides being more prevalent and more often preceded by MCI, is also characterised by a clinically homogenous phenotype dominated by attention and executive deficits^7,8^.

### Neuropsychological assessment

The neuropsychological assessment was carried out by means of a comprehensive battery of tests investigating different cognitive domains. The neuropsychological tests and their references can be found in Supplementary Tables S10 and S11.

In particular, among tests of episodic memory, we chose measures of delayed recall since they have been shown to have the greatest diagnostic accuracy in identifying the earliest cognitive changes of AD^125^.

MCI was diagnosed according to current consensus criteria of objective cognitive impairment on neuropsychological testing, essentially preserved daily functioning (i.e. intact basic activities of daily living (BADL) with no or minimal impairment of instrumental activities of daily living (IADL)) and no dementia^1^.

A cognitive domain was considered to be impaired if at least one test within that domain was impaired and MCI was classified in aMCI and naMCI based on the presence or absence of impairment in the episodic memory domain. Further subcategorisation in single- versus multiple-domain MCI was made for descriptive purposes only (Supplementary Table S12).

Further information on the diagnosis of MCI is given in the Supplementary Methods.

### Clinical assessment

All subjects received a full clinical assessment during which we collected general clinical information, as well as data on vascular risk and peripheral (i.e. non-cerebral) vascular burden. The former included composite vascular risk scores and the latter corresponded to the echographically-determined target organ damage (carotid atherosclerosis and LVH).

A more detailed description of the clinical assessment can be found in the Supplementary Methods.

### Assessment of cerebral vascular burden and atrophy

CT imaging was performed on a 64-slice CT scanner (OptimaCT660, GE Healthcare, Milwaukee, WI; tube voltage 120 KVp, tube current 150–350 mA with automatic modulation, slice thickness 0.625 mm, field of view (FOV) 250 × 250 mm^2^, matrix size 512 × 512). MRI was performed on a 3-T scanner (Achieva, Philips Medical Systems, Eindhoven, the Netherlands) using a standard acquisition protocol including the following sequences: (a) T1-weighted (relaxation time (TR) 9.9 ms, echo time (TE) 4.6 ms, flip angle (FA) 8°, slice thickness 1 mm , slice gap 0, FOV 240 × 240 mm^2^, matrix size 240 × 240), (b) T2-weighted turbo spin echo (TSE) (TR 2,491 ms, TE 77 ms, FA 90°, slice thickness 4 mm, slice gap 0, FOV 230 × 230 mm^2^ , matrix size 505 × 512), (c) Fluid-attenuated inversion recovery (FLAIR) (TR 11,000 ms, TE 125 ms, FA 90°, slice thickness 4 mm, slice gap 0, FOV 230 × 230 mm^2^, matrix size 249 × 344).

Cerebrovascular burden and atrophy of the insula and hippocampus were assessed on CT and MRI brain scans by means of standard semi-quantitative visual rating scales. DWML and periventricular white matter lesions (PVWML) were evaluated by means of a slightly modified version of Fazekas' scale^74^, scored on axial CT scans and on axial FLAIR MRI scans. Due to the acknowledged vascular (i.e. ischaemic) aetiology of DWML (see Supplementary Methods) the DWML score was used in the correlation analyses. HA was rated by means of Kim's 5-point scale^126^ on axial CT and axial T1-weighted MRI scans and by means of Scheltens' 5-point scale^127^ on coronal T1-weighted MRI scans. HA was rated separately for the right and left hemispheres and the average HA score was used in the correlation analyses since this measure has been shown to be particularly sensitive to AD^128^. IA was assessed on coronal T1-weighted MRI scans by means of the 4-point frontoinsula (FI) rating scale^76^, which was rated separately for the right and left hemispheres and then combined in an overall average score for use in the correlation analyses. All these scales are described in detail in the Supplementary Methods.

A single trained rater evaluated the anonymised scans of all participants. To provide independent validation of the visual ratings scored by the primary rater, a second rater, with expertise in the neuroradiological assessment of cognitive impairment, rated a random sample of brain scans (*n* = 50 CT, *n* = 50 MRI) from which inter-rater reliability was calculated . The primary rater also re-rated this subset of scans in order to compute intra-rater reliability. To improve rating consistency, raters were supplied with reference images of specific anatomic landmarks and illustrative examples of each rating scale. Ratings from the primary rater were used in the analyses. The assessment of reliability is illustrated in the Supplementary Methods.

Because of the clinical context of the study, although the vast majority of subjects underwent on-site neuroimaging, a very small number (*n* = 20: *n* = 7 aMCI, *n* = 10 naMCI and *n* = 3 CN) were scanned at other hospital sites due to personal logistical reasons. However, their neuroimaging data were acquired and included in the study since it is acknowledged that visual rating scales are robust to scanner differences (e.g.^129^).

The main neuroimaging tecnique in our study was CT, since CT brain scanning was performed in all MCI subjects. MRI scans were used to assess IA (since this was not possible on our conventional CT scans lacking coronal reconstructions) and in order to provide a gold-standard comparison with CT ratings of cerebrovascular burden and hippocampal atrophy (see “Discussion”).

### ApoE genotyping

Blood samples were drawn after at least 6 h of fasting and the ApoE genotype was determined, as described elsewhere^123^. Subjects were classified as ApoE4 carriers if they had at least one ApoE4 allele.

### Autonomic assessment

The autonomic assessment was carried out as previously described^20^. It was performed in a quiet room, with dimmed lighting and a comfortable temperature (22–24 °C), between 8:30 and 11:30 a.m. in order to minimise the effect of circadian changes in HRV. Participants were instructed to consume a light breakfast and refrain from caffeinated beverages, alcohol, smoking and vigorous physical activity in the 12 h prior to testing. After a standard 12-lead ECG, three-channel ECG recordings for HRV analysis were obtained by means of a digital Holter recorder (Spider View, Sorin Group Company).

The protocol was composed of two stages. The first (baseline) consisted of supine rest with free breathing: 15 min during which the subjects were asked to remain awake, silent and still, breathing spontaneously. The second (sympathetic stimulation) corresponded to an active standing manoeuvre: 10 min during which the subjects were asked to remain still and silent, breathing spontaneously, after standing upright in as smooth a motion as possible. To allow for stabilisation, only the last 5 min of each stage were analysed.

The spontaneous respiratory rate can be assessed by the peak frequency of the HF band on PSA (5-min average), but, for greater accuracy, it was also visually monitored (over five contiguous one-minute periods). Subjects with a respiratory rate < 9 breaths/min (< 0.15 Hz) or > 24 breaths/min (> 0.40 Hz) were excluded, due to the shift of the high frequency band which precludes proper interpretation of PSA^101^.

BP was recorded at the end of the baseline period using a validated digital spyghmomanometer over the brachial artery (OMRON M6).

At the end of the protocol participants were asked to rate their level of stress on a VAS scale from 0 (no stress) to 100 (maximum stress), since emotional stress can affect HRV^9^.

Unlike in our previous work^20^, we decided not to include in the protocol a stage of paced breathing at 12 breaths/min (parasympathetic stimulation)^80^. We are aware that a parasympathetic challenge could have provided more sensitive and direct information on the functioning of the parasympathetic nervous system, which would have been particularly valuable in our sample of naMCI who exhibit low/intermediate vascular risk (see “Exclusion criteria” and “Discussion”) and are thus likely to have mild frontal lobe vascular damage and slight parasympathetic dysfunction. Nevertheless, we chose to omit it because we believed it would introduce an intrinsic bias in the study. In fact, based on evidence of a strong relationship of higher-level complex everyday activities with executive functions^130^ and subcortical white matter damage^131^, it was reasonable to suppose that naMCI participants would have greater difficulty in correctly performing a cognitively demanding task like synchronising their breathing rhythm with pre-set acoustic signals from a metronome. Indeed, even if the appearance of a 0.2 Hz peak within the HF spectrum gives a rough confirmation that an individual has achieved the target respiratory rate within the 5-min analysis, it cannot rule out interference from short-term fluctuations in respiratory frequency.

### HRV analysis

HRV analysis was performed in the frequency domain (PSA) on 5-min ECG recordings from each of the two stages (baseline and active standing), in accordance with standard guidelines^10^. We used commercial software (Synescope version 3.10, Sorin Group Company) which conducts PSA by means of the Fast Fourier Transform (FFT), after linear interpolation/resampling at 4 Hz of the discrete event series (to obtain a regularly time-sampled signal) and filtering with a Hanning window (to attenuate leakage effects)^20^. The software corrects for ectopics by linear interpolation based on the surrounding sinus beats^9^. Although the software automatically detects non-sinus beats, the recordings were always manually overread by an experienced investigator, blinded to the subject's cognitive status, in order to ensure correct QRS complex classification and rhythm identification. Since there is no clear indication in the literature as to the amount of ectopic beats that it is acceptable to remove or remove and interpolate, we chose the most restrictive criterion of 1% of the total number of beats^20^. Therefore, recordings with excessive supraventricular or ventricular ectopy (i.e. ectopic beats > 1% of total beats) were excluded from analysis, as were those with other arrhythmias.

PSA can yield several different indices: VLF (≤ 0.04 Hz), LF (0.04–0.15 Hz), HF (0.15–0.4 Hz), TP (≤ 0.4 Hz), LFn, normalised HF (HFn) (corresponding respectively to LF/(LF + HF) × 100 and HF/(LF + HF) × 100) and the LF/HF ratio^9^ .

We chose to focus on LFn, the LF/HF ratio and HF as markers of autonomic function because other indices cannot be meaningfully interpreted. TP only quantifies overall autonomic modulation^9^. The nature of LF is highly controversial and it has been taken to reflect prevalently sympathetic modulation^9^, mixed sympathetic and parasympathetic modulation^9^, and predominantly parasympathetic modulation^89^. VLF is best evaluated over 24 h^132^ and its physiological underpinnings are still under discussion^133^. HFn was not included in the results since it is specularly correlated to LFn (given that they add up to a 100).

Although tranformed measures (LFn, HFn and the LF/HF ratio) have received ample support (e.g.^70,134,135^) and are extensively used in HRV studies across topics (e.g.^136-138^), they also suffer from limitations (e.g.^89,94^) which have been addressed in the Discussion. Within the context of autonomic reciprocity, LFn and the LF/HF ratio can be viewed as indices of sympathetic activation/parasympathetic withdrawal. HF is an acknowledged marker of parasympathetic activity^9,101^.

In particular, the changes of LFn, the LF/HF ratio and HF from baseline to active standing, assessed by Δ HRV indices (active standing measure—baseline measure) are especially sensitive markers of autonomic modulation because they directly quantify the response to orthostatic stress and explore the dynamic range of the ANS^70^.

Of the 183 subjects undergoing autonomic assessment 10 were excluded from HRV analysis (*n* = 4 for respiratory rate, *n* = 6 for excessive ectopic beats).

### Statistical analysis

Data are reported as mean (standard deviation) for continuous and ordinal variables and as number (percentage) for categorical variables. The normality of the data was assessed by using the Shapiro–Wilk test. HRV indices were normalised by logarithmic transformation to base 10 (lg), except for Δ HF which could not be normalised by transformations. The three groups were compared on categorical variables by means of the Chi-squared test or Fisher's exact test, and on continuous (and ordinal) variables by means of One-Way Analysis of Variance (ANOVA) or the Kruskall-Wallis test, as appropriate. All post-hoc pairwise comparisons were conducted with Bonferroni corrections. Analysis of Covariance (ANCOVA) was used to compare the three groups on HRV measures while controlling for potential confounders, which were chosen among the variables found to be significantly different across the groups, i.e. prevalence of hypertension, levels of physical activity and the STPI-T score. Use of ACE-I/ARB medications and the Cumulative Illness Rating Scale-morbidity (CIRS-m) score were not included in the ANCOVA in order to avoid loss of power due to multicollinearity since these variables were found, as expected, to be redundant with the prevalence of hypertension (Cramer's V = 0.774 and Eta squared = 0.209 respectively). For Δ HF a non parametric alternative to ANCOVA was used, i.e. rank-based regression (Rfit) from the "npsm" package in R^139^.

The relationship of autonomic function with relevant measures of cognition and of structural brain changes in each of the two MCI groups (aMCI and naMCI) was investigated by means of Spearman's correlation analysis, adjusted for prevalence of hypertension, levels of physical activity, STPI-T score and, in the case of measures of cognition, also demographics. Autonomic function was indexed by the HRV measures which were found to be significantly different across groups (Δ LFn, Δ LF/HF and standing HF); measures of cognition were the Z-scores for tests of memory (aMCI) as well as of attention and executive functioning (naMCI); measures of structural brain changes were the CT-based Fazekas' DWML (naMCI) and Kim's HA (aMCI) scores as well as the MRI-based FI rating score (aMCI). With regard to measures of cognition, we chose to compute a composite score for executive function, evaluated by as many as nine tests, to minimise the likelihood of type I error associated with multiple testing. Individual test scores were standardised by conversion to Z-scores and the average of the Z scores was taken to be the domain-specific Z-score. Z-scores for each test were calculated as (test score subject—mean score norm group)/standard deviation norm group, by using the mean and standard deviation of published normative data for raw scores (references available in Supplementary Table S10). Scores that quantified response time (i.e. Trail-Making Tests A and B ) or number of errors (i.e. Cognitive Estimates Total and Bizarre) were first multiplied by -1, so that negative Z-scores always indicated poor performance. For the sake of consistency and for ease of comparison, Z-scores were also generated in the same manner for each of two memory tests (prose-delayed recall and ROCF-delayed recall) and each of two attention tests (Bell Test and DCT). Since Z-score standardisation was based on normative data for the raw (i.e. non demographically-corrected) scores, demographics (i.e. age, gender and education) were also adjusted for in the correlation analyses, except for the Bell Test which requires no demographic adjustment (see relevant references in Supplementary Table S10).

To address the issue of inflation of type I error due to multiple comparisons between variables, we employed the Benjamini–Hochberg procedure^140^ with the False Discovery Rate (FDR) set at the conventional level for alpha (5%). This was preferred to the Bonferroni correction because the latter is known to be overly conservative in the case of highly correlated variables, like ours, leading to an undue loss in statistical power (i.e. an increase in false negatives), which would be inappropriate also given the exploratory nature of the study^141^.

The effect sizes for differences in HRV indices were calculated by comparing the aMCI group with the naMCI and CN groups, which were collapsed into a single group since they showed no significant differences in the autonomic response to standing. For the independent samples t-test, we used Cohen's d or Glass's delta depending on whether the assumption of homogeneity of variance was satisfied (as assessed by Levene's test)^142^. For Mann–Whitney's U-test, we used^143^ the correlation coefficient r. Effect sizes are reported in full in Supplementary Table S3.

Based on our previous finding of at least medium effect sizes^20^ and according to current guidelines for HRV studies^77^, we estimated that a minimum sample size of around 60 subjects per group would be required to achieve 80% statistical power at a 5% alpha level. Hence, our recruitment of more than 70 participants per group met (and exceeded) this minimum sample size recommendation.

A *P* value ≤ 0.05 was considered statistically significant. Analyses were performed by means of the statistical packages SPSS version 25.0 (SPSS Inc., Chicago, IL) and R version 3.5.2 (The R Foundation for Statistical Computing, Vienna, Austria) for Windows.

## Supplementary information

Supplementary information

## Data Availability

The datasets generated during and/or analysed during the current study are available from the corresponding author on reasonable request.
