# Supplementary material for: Autonomic function in amnestic and non-amnestic mild cognitive impairment: spectral heart rate variability analysis provides evidence for a brain–heart axis
Source: Sci Rep. 2020 Jul 15;10:11661. doi: 10.1038/s41598-020-68131-x (PMC7363846; doi:10.1038/s41598-020-68131-x)
Supplement: Supplementary file 1 — Supplementary information [file 41598_2020_68131_MOESM1_ESM.pdf]

Autonomic function in amnestic and non-amnestic mild cognitive impairment: spectral heart rate variability analysis provides evidence for a brain-heart axis

Paola Nicolini, Daniela Mari, Carlo Abbate, Silvia Inglese, Laura Bertagnoli, Emanuele Tomasini, Paolo D. Rossi, Federico Lombardi

Supplementary Information

Contents:

Supplementary Methods

MoCA screening

Exclusion criteria

Diagnosis of MCI

Clinical Assessment

Assessment of cerebral vascular burden

Assessment of hippocampal atrophy

Assessment of insular atrophy

Assessment of reliability

Supplementary Discussion

Predominance of female gender in the study sample

Differences between the aMCI and naMCI groups

Differences in neuropsychological tests

Differences in clinical characteristics

Differences in vascular burden

Limitations

Supplementary References

Supplementary Tables S1 to S12

Supplementary Methods

MoCA Screening

One main goal of the study was to increase the size of a previous sample of MCI subjects (*n*=40)[20], in order to allow its meaningful breakdown into two subgroups of aMCI and naMCI. To this purpose the MoCA was used as a screening tool, preliminary to enrollment, because of its high sensitivity for MCI, ranging from 90 to 97% in different studies[122,144,145], and its relatively broad-based assessment, allowing the detection of cognitive impairment even of the vascular type[146,147]. Given that the MoCA has a poor specificity for cognitive impairment, ranging from 35 to 60% in different studies[144,145], we expected that among subjects screening positive on the MoCA there would be a fair number of cognitively normal individuals that it would be reasonable to include in the study to also enhance the size of the control group. Therefore, subjects eligible for the study (see Exclusion criteria below) were screened with the MoCA at the standard threshold[122] and, if positive (i.e scoring below 26 points), were invited to undergo a comprehensive neuropsychological assessment for a definitive evaluation of cognitive status.

Thus, in both the current and previous enrollments all subjects received a comprehensive neuropsychological assessment, but in the current enrollment they were first screened with the MoCA.

Exclusion criteria

We applied the following exclusion criteria: a) non-sinus rhythm (atrial fibrillation and other arrhythmias, paced rhythms) since HRV analysis is by definition performed on sinus beats[9], b) clinical conditions with an established and significant effect on HRV: heart disease (coronary artery disease (CAD), heart failure (HF))[9], diabetes mellitus[9], neurological and psychiatric diseases (Parkinson's disease, stroke, major depression)[9,148] and severe diseases (respiratory, renal, autoimmune and neoplastic)[149], c) use of several cardioactive medications: beta-blockers, alpha-blockers, centrally-acting calcium-channel blockers (CCB), class I and III antiarrhytmic drugs, digoxin, d) use of several psychotropic medications: tricyclic antidepressants, selective serotonin-

noradrenaline reuptake inhibitors, atypical antidepressants, antipsychotics and cholinesterase inhibitors.

The choice of exclusion/inclusion criteria for medications has been extensively accounted for and referenced in our previous work[20].

Diagnosis of MCI

Raw neuropsychological test scores were converted to age-, gender- and education-adjusted scores based on published normative data for the Italian population (neuropsychological tests are referenced in Supplementary Table S10) and subjects were considered to be cognitively impaired if the adjusted score in at least one neuropsychological test fell below the 10th percentile of the normative score distribution. We selected the 10th percentile threshold, in accordance with several other Authors (e.g.[150-152]), because we believe that, relative to the 1 and 1.5 standard deviation cut-offs (i.e. the 7th and 16th percentiles), also commonly accepted[153], it provides a more appropriate balance between the risk of under- and over-diagnosing MCI.

Subcategorisation of aMCI and naMCI based on the number of domains affected (single domain (SD) versus multiple domain (MD)) was made for descriptive purposes only (see Supplementary Table S12).

A few issues deserve to be mentioned. First, we decided, consistently with other research groups (e.g.[150,152]) that subjective cognitive impairment was not required for a diagnosis of MCI; indeed this criterion is controversial since individuals with MCI are often unaware of cognitive deficits due to loss of insight[154], while cognitive complaints in normally functioning subjects may be associated with multiple factors including anxiety, depression, personality traits, physical health and ageing per se[155]. Second, the criterion of "no or minimal impairment in IADL" is recognised to be a challenging one, since it has no standard definition and is often operationalised through the use of clinical judgment[153,156]. Like other Authors[3,152,157,158], we regarded as minimally impaired those subjects who were dependent in just one IADL due to cognitive reasons[156]; we allowed impairments in BADL and IADL ascribed to non-cognitive disabilities (e.g. stress incontinence, movement limitation caused by arthritis)[150].

Clinical Assessment

We collected information on sociodemographics (age, gender, education), anthropometrics (Body Mass Index (BMI)), lifestyle habits (alcohol and coffee consumption, physical activity), blood tests for vascular risk (glucose and lipid panel), history of hypertension (defined as current antihypertensive drug therapy), comorbidity (CIRS-m score), medication history, functional status (BADL and IADL scores), global cognitive functioning (MMSE score) and psychological symptoms (STPI-T and GDS-s scores), as previously described and referenced[20].

Vascular risk factors were used to compute two validated and widely employed risk scores: the Framingham Stroke Risk Profile (FSRP)[159] and the Framingham General Cardiovascular Disease Risk Profile (FCVDRP)[160], which estimate the 10-year risk of stroke and cardiovascular events respectively. Incorporating individual risk factors in a composite risk score has a dual advantage: the score has greater predictive value since it is a weighted combination of risk factors meant to better capture their interactive effects, and dealing with a single summary measure minimises the risk of type I errors associated with multiple testing. Both scores were considered since they are in part complementary (e.g. the FSRP does not include total and HDL cholesterol but it does LVH). Cardiovascular risk factors and diseases which were exclusion criteria for the study (e.g. diabetes and atrial fibrillation) were scored zero points.

Peripheral (i.e. non-cerebral) vascular burden was evaluated based on echocardiographic evidence of carotid artery atherosclerosis and LVH. Subjects who did not have a recent (within the past year) echocardiogram or carotid Doppler scan were prescribed such exams.

Carotid atherosclerosis was determined by carotid IMT as well as by carotid plaque burden according to the NASCET (North American Symptomatic Carotid Endoarterectomy Trial) method[161]. Like other Authors (e.g.[162,163]), we recorded the maximum value for IMT and for percent stenosis occurring on either the left or right side because carotid atherosclerosis can be asymmetrical[164] and averaging bilateral values might lead to an underestimation of vascular damage.

LVH was defined as a left ventricular mass index (LVMI) > 95 and 115 g/m^2 in females and males respectively, based on current guidelines; as recommended, LVM was calculated from end-diastolic LV septum and posterior wall thicknesses and internal diameter by means of the cube formula and indexed for body surface area using the Mosteller formula[165].

Assessment of cerebral vascular burden

Cerebral vascular burden was assessed by evaluating WML. WML are defined as ill-defined, patchy areas of white matter rarefaction, which appear hypodense on CT and hyperintense on T2-weighted and FLAIR MRI sequences[166]. They are subcategorised as periventicular WML (PVWML) and DWML according to their location and are believed to have different origin. The former are immediately contiguous to the ventricular system and their aetiology is mainly neurodegenerative, related to the disruption of the ependymal layer, or mixed neurodegenerative/ischaemic; the latter are separated from the ventricles, within the subcortical white matter, and are due to chronic ischaemia[167,168].

Fazekas' scale is the most used and best validated visual scale for grading WML burden, can be applied to both CT and MRI, and is composed of two 4-point subscales which separately code the two subtypes of WML[169].

PVWML are rated as follows: 0=none, 1=caps or pencil-thin lining (i.e. small caps surrounding the frontal and occipital horns of the ventricles or thin bands along the borders of the ventricles), 2= smooth halo (i.e. thicker periventricular band) and 3=irregular lesions extending into the DWM.

DWML are rated as follows: 0=none or single punctate, 1=multiple punctate, 2=early confluence (connecting bridges) and 3=large confluence.

The scale requires assessment of axial CT or MRI images for the whole brain and is scored on the slice showing the most severe WML load.

Consistently with other Authors (e.g.[74,170,171]), we used a slightly modified version of Fazekas' scale which integrates more objective size criteria. This choice was made because CT imaging, prescribed to all participants, is recognised to be less sensitive than MRI to silent cerebrovascular disease and to have lower reliability[114], while it has been demonstrated that intra- and inter-rater agreement improves to the levels of MRI if more specific operational definitions are used[74]. In particular, we focused on potential major areas of ambiguity in the rating of neuroimages. With regard to PVWML, caps and bands were scored as 1 if < 5 mm , as 2 if between 5 and 10 mm and as 3 if they extended at a distance of > 10 mm from the ventricles (largest diameter measured parallel for caps and perpendicular for bands)[74,170]. With regard to DWML, since "early" and "large" confluence can sometimes be difficult to discriminate based on qualitative appearance[55], when such was the case DWML were scored as 2 or 3 according to the largest diameter of the area involved (between 10 and 25 mm and > 25 mm respectively)[74].

Assessment of hippocampal atrophy

HA is a sensitive biomarker of AD[115,153] and its radiological assessment is based on the evaluation of the thickness of the hippocampus and the degree of cerebrospinal fluid (CSF) accumulation in the fissures of the perihippocampal region[75,115]. Although HA assessment is more frequently performed by using Scheltens' scale[127] on coronal MRI images, there is consistent evidence in the literature, both indirect and direct, that it can also be carried out on axial CT scans. The indirect evidence comes from recent work from Korean research groups[118,126,172] that have shown substantial equivalence between Scheltens' coronal scale and Kim's axial scale on MRI scans, coupled with a large body of research demonstrating that the reliability of visual rating scales for HA is comparable on CT and MRI imaging[114,115,173]. The direct evidence comes from a number of older, CT-based, studies which have originally validated the use of CT axial scans for the rating of HA[75, 174-177] .

For CT scans and MRI scans HA was rated on a single axial slice, which showed the midbrain most prominently, by means of Kim's 5-point scale[126] where 0=no atrophy, 1=only widening of the perimesencephalic cistern, 2=also widening of the anterior temporal horn of the lateral ventricle, 3=also moderate loss of hippocampal volume (decreased width of hippocampus) and 4=severe loss of hippocampal volume.

For MRI scans, HA was also rated on a single coronal slice, located just behind the amygdala and mammillary bodies, in the area of the cerebral peduncles and cutting through the body of the hippocampus, by means of Scheltens' 5-point scale[127] where 0=no atrophy, 1= only widening of the choroid fissure, 2=also widening of the temporal horn of the lateral ventricle, 3=also moderate loss of hippocampal volume (decreased height of hippocampus) and 4=severe loss of hippocampal volume.

Assessment of insular atrophy

IA was assessed on three coronal MRI slices, the first anterior slice in which the anterior commissure was just visible as well as the two slices immediately posterior to it, by means of a 4-point frontoinsula (FI) rating scale focusing on the widening of the circular sulcus (CS)[76]. The scale is graded as follows: 0=no atrophy (closed CS), 1=mild widening of the CS (CSF just visible), 2=moderate widening of the CS (with emergence of an arrow head shape pointing towards the midline), 3=severe widening along the length of the CS (which takes on a triangular shape), and the score is averaged over the three slices. The scale is designed for the assessment of coronal images

and thus could be applied only to the MRI scans (T1-weighted sequences) since the CT scans in our study were conventional ones, lacking coronal reconstructions.

Assessment of reliability

Intra- and inter-rater reliability for visual rating scales were determined by using Cohen's weighted kappa (K_w), which is the preferred measure for ordinal scales since it takes into account the degree of discordance in ratings and differentially penalises disagreements according to their magnitude (i.e. ratings that are further apart on the ordinal scale are "weighted" so as to carry more importance)[178]. To facilitate comparison with other studies (e.g.[76]), we also calculated the intraclass correlation coefficient (ICC), specifically a two-way random, absolute, single-rater model[179]. We used a random sample of 50 scans (*n*=50 CT and *n*=50 MRI) based on a general heuristic rule that a sample of at least 30 subjects is required for the evaluation of reliability[180] and on the formula by Cicchetti[181] that yields a minimum sample size of *n*=30 or *n*=50 respectively for an ordinal scale with 5 (the HA scale) or 4 (the WML and IA scales) categories.

Supplementary Discussion

Predominance of female gender in the study sample

The largely female composition of the sample is likely to be related to the longer life expectancy of women[2], to the role of female gender as a risk factor for AD[182] and to the association of male gender with (excluded) overt cardio- and cerebrovascular disease[159,160].

Differences between the aMCI and naMCI groups

Differences in neuropsychological tests

On neuropsychological assessment the aMCI group showed the worst performance in episodic memory tests, as expected per classification criteria, but also in the test of category fluency (Supplementary Table S11). There is an increasing consensus in the literature that impaired access to semantic information occurs early in the trajectory of AD, reflecting involvement of the temporal lobes, and that category fluency is a sensitive measure of AD at the MCI[183-185] and even preclinical[186] stage.

There was no significant difference between the aMCI and naMCI groups in other tests, including those gauging attention and executive functioning. This can be attributed to the high prevalence within the aMCI group of the MD subtype (SD vs MD 15.9 % vs 84.1% , see Supplementary Table S12), a finding which is shared by other studies (e.g.[104, 187]). Although this may in part be due to the

use of a neuropsychological battery that extensively covers non-memory domains, it can also reflect the natural history of AD. In fact, neuropathology is known to rapidly spread from the limbic system to the neocortex to yield a multiple cognitive systems breakdown that has been described in the pre-dementia[107] and even in the pre-symptomatic[188] stage of the disease.

In the naMCI group, the pattern of cognitive deficits, showing predominant executive dysfunction (91.4%) and frequent impairment in attention, visuospatial skills and language (41.9%, 38.7% and 37.6% respectively), is consistent with the neuropsychological profile of vascular cognitive impairment[189-191]. Taken together, these results also explain why aMCI subjects exhibited the worst performance on tests of global cognitive functioning like the MMSE and MoCA.

Differences in clinical characteristics

The naMCI group had a greater prevalence of hypertension and higher scores on the STPI-T scale measuring trait anxiety while physical activity was lower in the aMCI group(see main text Table 1).

The greater prevalence of hypertension in the naMCI group fits in with the greater degree of target organ damage (LVMI, carotid plaque and DWML), but should not be taken as implying that hypertension was not controlled. In fact all hypertensive participants were treated and, although the study was not designed to assess blood pressure (BP) control since it lacks 24-hour ambulatory monitoring, it enrolled older adults accessing secondary care whose BP is likely to have been adequately managed by their GPs. Indeed, there is evidence that well-controlled hypertension is associated with a high prevalence of LVH (35-52%)[192,193] with carotid plaque progression[194] and with brain changes[195], especially in older subjects in whom more prolonged exposure to high BP may have led to more advanced (and thus irreversible) structural damage[194,196,197].

The higher levels of trait anxiety exhibited by naMCI subjects are in line with the notion that symptoms of anxiety are common in vascular cognitive impairment[198] and are thought to have their neural basis in a hypoactive prefrontal cortex which results in hyperactivation of the amygdala[199]. Since trait anxiety refers to an individual's general disposition to experience anxiety (i.e. it is a relatively stable personality trait)[200], it is not surprising that VAS-stress scores do not differ among groups as they measure another aspect of anxiety, which is a transient emotional state relative to a specific situation.

The lower physical activity in the aMCI group is in agreement with recent reports that physical activity has a protective effect on AD but not on VAD[201,202], although reverse causation could also be involved[203].

Differences in vascular burden

Although there was no difference in the composite cardiovascular risk scores between aMCI and naMCI subjects (see main text Table 1), the latter exhibited, as expected, greater vascular burden, both peripheral (i.e. carotid atherosclerosis and LVMI) and cerebral (i.e. DWML) (see main text Tables 2 and 3). This observation is in agreement with the general understanding that atherosclerosis and LVH are multifactorial and protracted phenomena resulting from the complex interplay of a number of factors (including genetic predisposition, the hormonal milieu and the duration of exposure) which cannot be adequately captured by traditional risk algorithms[204,205]. Indeed, several studies have reported either no association between MCI subtypes and vascular risk factors[8,206,207] or a prevalent association with "hard" risk factors such as CAD, stroke and diabetes[208,209], which were exclusion criteria for our study.

Notably, we found that plaque burden was significantly higher in the naMCI group whereas IMT was only marginally increased, which fits in well with the largely held view that they quantify different pathological changes. In fact, IMT is primarily associated with smooth muscle hypertrophy/hyperplasia due to BP- and age-induced shear stress, while plaque truly reflects the atherosclerotic process (e.g.[210]). We also found that the LVMI was higher in the naMCI group, although the prevalence of LVH was only borderline so. This speaks of the greater sensitivity to cardiovascular risk of a continuous, rather than a binary, variable[211], especially in a population where several factors associated with LVH (such as diabetes, cardiac and kidney disease) have been excluded[205].

Also, the visual rating score for cerebral DWML was significantly higher in the naMCI group on both CT and MRI scans. The PVWML score, instead, was higher in the aMCI group only on the CT scans. The CT findings are consonant with the presumed different aetiology (ischaemic vs neurodegenerative) of such lesions[151,167,168]. The lack of a significant difference across groups in PVWML on MRI stems from an increased detection of PVWML by MRI in the groups with lower PVWML burden (naMCI and CN). This is likely to be a consequence of the greater sensitivity of MRI for WML in general[114] and of FLAIR sequences for PVWML in particular (since greater suppression of the CSF signal provides better contrast between the CSF and bordering WML)[212,213].

Limitations

The fact that neuroimaging was unavailable for some subjects could potentially lead to a selection bias. Nevertheless, in CN subjects undergoing CT scans, bias would likely be operating against the finding of significant differences in brain atrophy and vascular burden across groups, in that it

would select individuals at higher risk of cognitive impairment, making our results more conservative. Also, if CN subjects are removed from the analyses of between-group WML load and HA on CT, the comparisons between aMCI and naMCI subjects retain their statistical significance. As far as MRI scans are concerned, it could be supposed that MCI subjects who were prescribed MRI scanning were those with worse cognitive deficits, resulting in an amplification of contrasts in brain MRI parameters across the three groups. When comparing MCI subjects with and without MRI scans (Supplementary Tables S6 and S7), we found no significant differences in the clinical and imaging characteristics between the two groups in aMCI subjects, but naMCI subjects with MRI scans had indeed a worse MMSE. However, it should be borne that in mind that the correlation analyses are not prone to be affected, since in the naMCI group they included only CT parameters, while a single MRI parameter (IA) was considered solely in the aMCI group.

Also, one could object that currently enrolled CN subjects (based on a positive MoCA screening followed by neuropsychological assessment) were cognitively more impaired than those enrolled earlier (based only on neuropsychological assessment). However, we found no substantial differences in the clinical, neuropsychological and imaging variables of the two groups of CN subjects (Supplementary Table S8), in keeping with the know low specificity of the MoCA for cognitive impairment (see Supplementary Methods).

Supplementary References

144. Luis, C. A., Keegan, A. P. & Mullan, M. Cross validation of the Montreal Cognitive Assessment in community dwelling older adults residing in the Southeastern US. *Int J Geriatr Psychiatry*. **24**, 197-201 (2009).
145. Larner, A. J. Screening utility of the Montreal Cognitive Assessment (MoCA): in place of--or as well as--the MMSE? *Int Psychogeriatr*. **24**, 391-396 (2012).
146. Koski, L. Validity and applications of the Montreal cognitive assessment for the assessment of vascular cognitive impairment. *Cerebrovasc Dis*. **36**, 6-18 (2013).
147. Dichgans, M. & Leys, D. Vascular Cognitive Impairment. *Circ Res*. **120**, 573-591 (2017).
148. Kemp, A. H. et al. Impact of depression and antidepressant treatment on heart rate variability: a review and meta-analysis. *Biol Psychiatry*. **67,** 1067-1074 (2010).
149. Uusitalo, A. L. et al. Role of genetic and environmental influences on heart rate variability in middle-aged men. *Am J Physiol Heart Circ Physiol*. **293,** H1013-1022 (2007).
150. Solfrizzi, V. et al. Italian Longitudinal Study on Aging Working Group. Vascular risk factors, incidence of MCI, and rates of progression to dementia. *Neurology*. **63**, 1882-1891 (2004).

151. Delano-Wood, L. et al. Heterogeneity in mild cognitive impairment: differences in neuropsychological profile and associated white matter lesion pathology. *J Int Neuropsychol Soc*. **15,** 906-914 (2009).

152. Knopman, D. S. et al. Mild Cognitive Impairment and Dementia Prevalence: The Atherosclerosis Risk in Communities Neurocognitive Study (ARIC-NCS). *Alzheimers Dement (Amst)*. **2**, 1-11 (2016).

153. Albert, M. S. et al. The diagnosis of mild cognitive impairment due to Alzheimer's disease: recommendations from the National Institute on Aging-Alzheimer's Association workgroups on diagnostic guidelines for Alzheimer's disease. *Alzheimers Dement*. **7**, 270-279 (2011).

154. Goerlich, K.S et al. Neuroanatomical and Neuropsychological Markers of Amnestic MCI: A Three-Year Longitudinal Study in Individuals Unaware of Cognitive Decline. *Front Aging Neurosci*. **9**, 34 (2017).

155. Rabin, L. A., Smart, C. M. & Amariglio, R. E. Subjective Cognitive Decline in Preclinical Alzheimer's Disease. *Annu Rev Clin Psychol*. **13**, 369-396 (2017).

156. Petersen, R. C. Mild cognitive impairment as a diagnostic entity. *J Intern Med*. **256,** 183-194 (2004).

157. Inzitari, D. et al. Risk of rapid global functional decline in elderly patients with severe cerebral age-related white matter changes: the LADIS study. *Arch Intern Med*. **167**, 81-88 (2007).

158. Luck, T. et al. Impact of impairment in instrumental activities of daily living and mild cognitive impairment on time to incident dementia: results of the Leipzig Longitudinal Study of the Aged. *Psychol Med*. **41**, 1087-1097 (2011).

159. D'Agostino, R. B., Wolf, P. A., Belanger, A. J. & Kannel, W. B. Stroke risk profile: adjustment for antihypertensive medication. The Framingham Study. *Stroke.* **25**, 40-3 (1994).

160. D'Agostino, R. B. Sr et al. General cardiovascular risk profile for use in primary care: the Framingham Heart Study. *Circulation*. **117**, 743-753 (2008).

161. Oates, C. P. et al. Joint recommendations for reporting carotid ultrasound investigations in the United Kingdom. *Eur J Vasc Endovasc Surg*. **37**, 251-261 (2008).

162. Notsu, Y. et al. Association of High-Density Lipoprotein Subclasses with Carotid Intima-Media Thickness: Shimane CoHRE Study. *J Atheroscler Thromb*. **25**, 42-54 (2018).

163. O'Leary, D. H. et al. Distribution and correlates of sonographically detected carotid artery disease in the Cardiovascular Health Study. The CHS Collaborative Research Group. *Stroke*. **23**, 1752-1760 (1992).

164. Rosfors, S., Hallerstam, S., Jensen-Urstad, K., Zetterling, M. & Carlström, C. Relationship between intima-media thickness in the common carotid artery and atherosclerosis in the carotid bifurcation. *Stroke*. **29**, 1378-1382 (1998).

165. Lang, R. M. et al. Recommendations for cardiac chamber quantification by echocardiography in adults: an update from the American Society of Echocardiography and the European Association of Cardiovascular Imaging. *J Am Soc Echocardiogr*. **28**, 1-39.e14 (2015) .

166. Smith, E. E. et al. American Heart Association Stroke Council; Council on Cardiovascular Radiology and Intervention; Council on Functional Genomics and Translational Biology; and Council on Hypertension. Prevention of Stroke in Patients With Silent Cerebrovascular Disease: A Scientific Statement for Healthcare Professionals From the American Heart Association/American Stroke Association. *Stroke*.**48**, e44-e71 (2017).

167. Schmidt, R. et al. Heterogeneity in age-related white matter changes. *Acta Neuropathol*. **122**, 171-185 (2011).

168. Lin, J., Wang, D., Lan, L. & Fan, Y. Multiple Factors Involved in the Pathogenesis of White Matter Lesions. *Biomed Res Int*. **2017**, 9372050 (2017).

169. Fazekas, F., Chawluk, J. B., Alavi, A., Hurtig, H. I. & Zimmerman, R. A. MR signal abnormalities at 1.5 T in Alzheimer's dementia and normal aging. *AJR Am J Roentgenol*. **149**, 351-356 (1987).

170. Sargento-Freitas, J. et al. Different locations but common associations in subcortical hypodensities of presumed vascular origin: cross-sectional study on clinical and neurosonologic correlates. *BMC Neurol*. **14**, 24 (2014).

171. Pantoni, L. et al. Effect of Attention Training in Mild Cognitive Impairment Patients with Subcortical Vascular Changes: The RehAtt Study. *J Alzheimers Dis*. **60**, 615-624 (2017).

172. Jang, J. W. et al. Alzheimer’s Disease Neuroimaging Initiative. A 'Comprehensive Visual Rating Scale' for predicting progression to dementia in patients with mild cognitive impairment. *PLoS One*. **13**, e0201852 (2018).

173. Wattjes, M. P. et al. Diagnostic imaging of patients in a memory clinic: comparison of MR imaging and 64-detector row CT. *Radiology*. **253,** 174-183 (2009).

174. Soininen, H., Puranen, M. & Riekkinen, P. J. Computed tomography findings in senile dementia and normal aging. *J Neurol Neurosurg Psychiatry*. **45**, 50-54 (1982).

175. De Leon, M. J. et al. The radiologic prediction of Alzheimer disease:the atrophic hippocampal formation. *AJNR Am J Neuroradiol*. **14**, 897-906 (1993).

176. De Leon, M. J. et al. Frequency of hippocampal formation atrophy in normal aging and Alzheimer's disease. Neurobiol *Aging*. **18**, 1-11 (1997).

177. Frisoni, G. B. et al. Radial width of the temporal horn: a sensitive measure in Alzheimer disease. *AJNR Am J Neuroradiol*. **23**, 35-47 (2002).
178. Cohen, J. Weighted kappa: nominal scale agreement with provision for scaled disagreement or partial credit. *Psychol Bull*. **70**, 213-20 (1968).
179. Shrout, P. E. & Fleiss, J. L. Intraclass correlations: uses in assessing rater reliability. *Psychol. Bull*. **86**, 420-428 (1979).
180. McHugh, M. L. Interrater reliability: the kappa statistic. *Biochem Med (Zagreb)*. **22**, 276-282 (2012).
181. Cicchetti, D. V. Testing the Normal Approximation and Minimal Sample Size Requirements of Weighted Kappa When the Number of Categories is Large. *Applied Psychological Measurement*. **5**, 101–104 (1981).
182. Congdon, E. E. Sex Differences in Autophagy Contribute to Female Vulnerability in Alzheimer's Disease. *Front Neurosci*. **12,** 372 (2018).
183. Saxton, J. al. Preclinical Alzheimer disease: neuropsychological test performance 1.5 to 8 years prior to onset. *Neurology*. **63**, 2341-2347 (2004).
184.Wilson, R. S., Leurgans, S. E., Boyle, P. A. & Bennett, D. A. Cognitive decline in prodromal Alzheimer disease and mild cognitive impairment. *Arch Neurol*. **68**, 351-356 (2011).
185. Mura, T. et al. Measuring cognitive change in subjects with prodromal Alzheimer's disease. J *Neurol Neurosurg Psychiatry*. **85**, 363-370 (2014).
186. Papp, K. V., Rentz, D. M., Orlovsky, I., Sperling, R. A. & Mormino, E. C. Optimizing the preclinical Alzheimer's cognitive composite with semantic processing: The PACC5. *Alzheimers Dement (NY)*. **3**, 668-677 (2017).
187. Rapp, S. R. et al. Subtypes of mild cognitive impairment in older postmenopausal women: the Women's Health Initiative Memory Study. *Alzheimer Dis Assoc Disord*. **24**, 248-255 (2010).
188. Mistridis, P., Krumm, S., Monsch, A. U., Berres, M. & Taylor, K. I. The 12 Years Preceding Mild Cognitive Impairment Due to Alzheimer's Disease: The Temporal Emergence of Cognitive Decline. *J Alzheimers Dis*. **48**, 1095-1107 (2015).
189. Clark, L. R. et al. Are empirically-derived subtypes of mild cognitive impairment consistent with conventional subtypes? *J Int Neuropsychol Soc*. **19**, 635-645 (2013).
190. Sudo, F. K. et al. Neuroimaging criteria and cognitive performance in vascular mild cognitive impairment: A systematic review. *Dement Neuropsychol*. **9**, 394-404 (2015).
191. Banerjee, G. et al. Total MRI Small Vessel Disease Burden Correlates with Cognitive Performance, Cortical Atrophy, and Network Measures in a Memory Clinic Population. *J Alzheimers Dis*. **63**, 1485-1497 (2018).

192. Jones, E., Morgan, T. O., Califiore, P. & Johns, J. Prevalence of left ventricular hypertrophy in elderly patients with well controlled hypertension. *Clin Exp Pharmacol Physiol.* **17**, 207-210 (1990).

193. Sole-Cabrera, R. et al. Left ventricular hypertrophy is more important when higher is central systolic blood pressure in mild to moderate hypertensive patients. *J Hypertens*. **33,** e164-165 (2015).

194. Izzo, R. et al. Development of new atherosclerotic plaque in hypertensive patients: an observational registry study from the Campania-Salute network. *J Hypertens*. **33**, 2471-6 (2015).

195. Salerno, J. A. et al. Brain atrophy in hypertension. A volumetric magnetic resonance imaging study. *Hypertension*. **20,** 340-348 (1992).

196. Strandgaard, S. Autoregulation of cerebral blood flow in hypertensive patients. The modifying influence of prolonged antihypertensive treatment on the tolerance to acute, drug-induced hypotension. *Circulation*. **53**, 720-727 (1976).

197. Mancia, G. et al. Pressioni Arteriose Monitorate E Loro Associazioni (PAMELA) Study. Prevalence of left ventricular hypertrophy in hypertensive patients without and with blood pressure control: data from the PAMELA population. Pressioni Arteriose Monitorate E Loro Associazioni. *Hypertension*. **39**, 744-749 (2002).

198. Seignourel, P. J., Kunik, M. E., Snow, L., Wilson, N. & Stanley, M. Anxiety in dementia: a critical review. *Clin Psychol Rev*. **28**, 1071-1082 (2008).

199. Ironside, M. et al. Effect of Prefrontal Cortex Stimulation on Regulation of Amygdala Response to Threat in Individuals With Trait Anxiety: A Randomized Clinical Trial. *JAMA Psychiatry*. **76**, 71-78 (2018).

200. Spielberger, C.D., Gorsuch, R. L., Lushene, R. E., Vagg, P. R & Jacobs, G. A. *Manual For The State-Trait Anxiety Inventory* (Consulting Psychologist Press, 1983).

201. Guure, C. B., Ibrahim, N. A., Adam, M. B. & Said, S. M. Impact of Physical Activity on Cognitive Decline, Dementia, and Its Subtypes: Meta-Analysis of Prospective Studies. *Biomed Res Int*. **2017**, 9016924 (2017).

202. Xu, W. et al. Leisure time physical activity and dementia risk: a dose-response meta-analysis of prospective studies. *BMJ Open*. **7**, e014706 (2017).

203. Sabia, S. et al. Physical activity, cognitive decline, and risk of dementia: 28 year follow-up of Whitehall II cohort study. *BMJ*. **357**, j2709 (2017).

204. Waldstein, S. R. & Katzel, L. I. Considering Cumulative Risk for Cardiovascular Disease in Relation to Cognitive Function: a Comment on DeRight et al. *Ann Behav Med*. **49**, 305-306 (2015).

205. Aronow, W. S. Hypertension and left ventricular hypertrophy. *Ann Transl Med*. **5**, 310 (2017).
206. Van de Pol, L. A. et al. White matter hyperintensities and medial temporal lobe atrophy in clinical subtypes of mild cognitive impairment: the DESCRIPA study. *J Neurol Neurosurg Psychiatry*. **80**, 1069-1074 (2009).
207. Dufouil, C. et al. MEMENTO cohort Study Group. Cognitive and imaging markers in non-demented subjects attending a memory clinic: study design and baseline findings of the MEMENTO cohort. *Alzheimers Res Ther*. **9**, 67 (2017).
208. Mariani, E. et al. Vascular risk factors in mild cognitive impairment subtypes. Findings from the ReGAl project. *Dement Geriatr Cogn Disord*. **24**, 448-456 (2007).
209. He, J. et al. Differences in brain volume, hippocampal volume, cerebrovascular risk factors, and apolipoprotein E4 among mild cognitive impairment subtypes. *Arch Neurol.***66**, 1393-1399 (2009).
210. Mathiesen, E. B et al. Carotid plaque area and intima-media thickness in prediction of first-ever ischemic stroke: a 10-year follow-up of 6584 men and women: the Tromsø Study. *Stroke*. **42**, 972-978 (2011).
211. Foppa, M., Duncan, B. B. & Rohde, L. E. Echocardiography-based left ventricular mass estimation. How should we define hypertrophy? *Cardiovasc Ultrasound*. **17**, 3:17 (2005).
212. Piguet, O. et al. Comparing white matter lesions on T2 and FLAIR MRI in the Sydney Older Persons Study. *Eur J Neurol*. **12**, 399-402 (2005).
213. Haller, S. et al. Do brain T2/FLAIR white matter hyperintensities correspond to myelin loss in normal aging? A radiologic-neuropathologic correlation study. *Acta Neuropathol Commun*. **1**,14 (2013).
214. Vallar, G., Rusconi, M. L., Fontana, S. & Musicco, M. Three clinical tests for the assessment of visuospatial exploration. Norms from 212 normal subjects. *Archivio di Psicologia, Neurologia e Psichiatria*. **54,** 827-41 (1994).
215. Spinnler, H. & Tognoni, G. Italian standardization and classification of Neuropsychological tests. The Italian Group on the Neuropsychological Study of Aging. *J Neurol Sci*. **8,** 1-120 (1987).
216. Carlesimo, G.A. et al. Normative data of two memory tasks: Short-Story recall and Rey's Figure. *Nuova Rivista di Neurologia*. **12,** 1-13 (2002).
217. Orsini, A. et al. Verbal and spatial immediate memory span: normative data from 1355 adults and 1112 children. *Ital J Neurol Sci*. **8,** 539-548 (1987).
218. Monaco, M., Costa, A., Caltagirone, C. & Carlesimo, G. A. Forward and backward span for verbal and visuo-spatial data: standardization and normative data from an Italian adult population. *Neurol Sci*. **34,** 749-754 (2013).

219. Giovagnoli, A. R. et al. Trail making test: normative values from 287 normal adult controls. *Ital J Neurol Sci.***17,** 305-309 (1996).
220. Della Sala, S., MacPherson, S. E., Phillips, L. H., Sacco, L. & Spinnler, H. How many camels are there in Italy? Cognitive estimates standardised on the Italian population. *Neurol Sci*. **24,** 10-15 (2003).
221. Novelli, G. et al. Three clinical tests for the assessment of verbal long-term memory function: Norms from 320 normal subjects. *Archivio di Psicologia, Neurologia e Psichiatria*. **47,** 278-296 (1986).
222. Laiacona, M., Barbarotto, R., Trivell, C. & Capitani, E. Dissociazioni semantiche e intercategoriali: descrizione di una batteria standardizzata e dati normativi. *Arch Psicol Neurol Psichiatr*. **54,** 209-248 (1993).
223. Caffarra, P., Vezzadini, G., Dieci, F., Zonato, F. & Venneri, A. Rey-Osterrieth complex figure: normative values in an Italian population sample. *Neurol Sci*. **22,** 443-447 (2002).
224. De Renzi, E., Motti, F. & Nichelli, P. Imitating gestures. A quantitative approach to ideomotor apraxia. Arch Neurol. **37,** 6-10 (1980).

Supplementary Tables

Supplementary Table S1 Cerebrovascular burden and hippocampal atrophy on magnetic resonance imaging in the study groups

	CN (*n*=78) †	aMCI (*n*=82) ‡	naMCI (*n*=93) ¶	*P*-value
Fazekas' scale score				
PVWML	1.6 (0.7)	1.7 (0.8)	1.6 (0.7)	0.707
DWML	1.4 (0.5)	1.4 (0.8)	1.9 (0.8)	0.004 [a**]
Scheltens' scale score				
Right HA (coronal)	1.8 (0.8)	2.4 (0.9)	1.5 (0.6)	< 0.001 [a***]
Left HA (coronal)	1.9 (0.8)	2.6 (0.9)	1.8 (0.6)	< 0.001 [a ***,b*]
Mean HA (coronal)	1.9 (0.8)	2.5 (0.8)	1.7 (0.5)	< 0.001 [a***]
Kim's scale score				
Right HA (axial)	1.9 (1.0)	2.7 (1.0)	1.6 (0.7)	<0.001 [a***]
Left HA (axial)	1.9 (1.0)	2.6 (0.9)	1.9 (0.7)	<0.001 [a***,b*]
Mean HA (axial)	2.0 (1.0)	2.6 (0.9)	1.7 (0.7)	<0.001 [a***]

Legend

Scale scores expressed as mean (standard deviation). Kruskall-Wallis test with Bonferroni-corrected pairwise comparisons. † available for *n*= 14, ‡ available for *n*=54,¶ available for *n*=56. [a] significant difference between aMCI and naMCI, [b] significant difference between aMCI and CN, [c] significant difference between naMCI and CN, *** $p \leq 0.001$, ** $p < 0.01$, * $p < 0.05$. Abbreviations: CN, cognitively normal (controls); aMCI, amnestic mild cognitive impairment; naMCI, non-amnestic mild cognitive impairment; PVWML, periventricular white matter lesions; DWML, deep white matter lesions; HA, hippocampal atrophy.

Supplementary Table S2 Intra- and inter-rater reliability for visual rating scales on neuroimaging

	Weighted Kappa (K_w)		Intraclass correlation coefficient (ICC)	
	Intra-rater	Inter-rater	Intra-rater	Inter-rater
Computed tomography (CT)				
PVWML	0.972	0.812	0.982	0.879
DWML	0.966	0.780	0.973	0.831
Right HA	0.885	0.778	0.940	0.885
Left HA	0.961	0.902	0.979	0.948
Magnetic Resonance Imaging (MRI)				
PVWML	0.935	0.840	0.960	0.897
DWML	1.000	0.777	1.000	0.853
Right HA (coronal)	0.948	0.853	0.966	0.908
Left HA (coronal)	0.923	0.808	0.952	0.879
Right HA (axial)	0.901	0.823	0.947	0.904
Left HA (axial)	0.920	0.790	0.954	0.885
Right IA	0.880	0.808	0.963	0.946
Left IA	0.827	0.795	0.916	0.913

Legend

Reliability evaluated on a random sample of scans (*n*=50 CT and *n*=50 MRI). ICC is a two-way random, absolute, single-rater model. Abbreviations: PVWML, periventricular white matter lesions DWML, deep white matter lesions; HA, hippocampal atrophy; IA, insular atrophy. Fazekas' scale for DWML and PVWML on axial CT and MRI; Kim's scale for HA on axial CT and MRI; Scheltens' scale for HA on coronal MRI; Frontoinsula rating scale for IA on coronal MRI.

Supplementary Table S3 Effect sizes for HRV indices

	aMCI (*n*=82)	naMCI + CN (*n*=171)	*P*-value	Effect Size
RR interval (ms)				
Baseline	941.5 (166.4)	923.0 (131.8)	0.436 [a]	0.103 [c]
Standing	886.8 (151.2)	870.2 (125.4)	0.456 [a]	0.098 [c]
Δ Standing	-54.7 (102.4)	-52.8 (73.5)	0.209 [a]	0.140 [c]
LFn (n.u)				
Baseline	61.9 (17.2)	59.7 (15.3)	0.500 [a]	0.089 [c]
Standing	62.9 (18.5)	75.5 (13.4)	< 0.001 [a]	1.042 [d]
Δ Standing	1.0 (11.6)	15.8 (10.9)	< 0.001 [a]	2.546 [d]
LF/HF				
Baseline	2.5 (2.3)	2.0 (1.5)	0.234 [a]	0.155 [c]
Standing	2.8 (2.7)	4.7 (3.7)	< 0.001 [a]	0.833 [d]
Δ Standing	0.3 (2.2)	2.7 (2.9)	< 0.001 [a]	0.895 [c]
HF (ms^2)				
Baseline	288.1 (529.6)	194.0 (303.5)	0.408 [a]	0.109 [c]
Standing	278.0 (575.6)	87.1 (150.3)	< 0.001 [a]	0.566 [c]
Δ Standing	-10.1 (227.7)	-106.9 (220.4)	< 0.001 [b]	0.220 [e]
TP (ms^2)				
Baseline	1605.2 (1698.2)	1411.7 (1603.0)	0.199 [a]	0.174 [c]
Standing	1438.9 (1583.4)	1134.3 (1559.4)	0.197 [a]	0.190 [d]
Δ Standing	-166.3 (1531.6)	-277.4 (1717.4)	0.494 [a]	0.103 [c]
VLF (ms^2)				
Baseline	944.9 (1145.3)	925.7 (1155.3)	0.586 [a]	0.074 [c]
Standing	794.6 (742.1)	795.5 (1280.2)	0.570 [a]	0.076 [c]
Δ Standing	-150.3 (1230.5)	-130.3 (1580.8)	0.571 [a]	0.087 [c]
LF (ms^2)				
Baseline	372.2 (568.8)	292.0 (533.9)	0.078 [a]	0.239 [c]
Standing	366.2 (594.2)	251.8 (400.0)	0.422 [a]	0.125 [d]
Δ Standing	-5.9 (367.6)	-40.2 (285.1)	0.449 [a]	0.117 [c]

Legend

HRV indices expressed as mean (standard deviation). [a] Student's t-test for independent samples on log_{10}-transformed values, [b] Mann-Whitney's U-test on untransformed values (normalisation not achieved with log_{10}-transformation), [c] Cohen's d, [d] Glass's delta (with standard deviation of the naMCI + CN group), [e] Correlation coefficient r. Cohen's d and Glass's delta should be interpreted as small (0.25 to 0.5), medium (0.5 to 0.9) and large (> 0.9) according to current recommendations for HRV[77]. Correlation coefficient r can be interpreted as small (0.1 to 0.3), medium (0.3 to 0.5) and large (> 0.5) according to Cohen's standard guidelines[81]. Abbreviations: aMCI, amnestic mild cognitive impairment; naMCI, non-amnestic mild cognitive impairment; CN, cognitively normal; n.u, normalised units; LFn, low frequency power (normalised); LF/HF, ratio of low frequency power (LF) to high frequency power (HF); TP, total power; VLF, very low frequency power; Δ standing, standing HRV index - baseline HRV index.

Supplementary Table S4 Other HRV indices in the study groups

	CN (*n*=78)	aMCI (*n*=82)	naMCI (*n*=93)	*P*-value (ANOVA)	*Q*	*P*-value (ANCOVA)	*Q*
Baseline TP (ms^2)	1681.9 (1964.4)	1605.2 (1698.2)	1185.1 (1185.2)	0.077	0.284	0.076	0.281
Standing TP (ms^2)	1217.9 (1876.3)	1438.9 (1583.4)	1064.2 (1239.3)	0.394	0.506	0.130	0.281
Δ TP (ms^2)	-464.0 (2154.6)	-166.3 (1531.6)	-120.9 (1229.0)	0.144	0.284	0.184	0.281
Baseline VLF (ms^2)	1165.5 (1479.8)	944.9 (1145.3)	724.6 (737.4)	0.071	0.284	0.136	0.281
Standing VLF (ms^2)	894.6 (1561.2)	794.6 (742.1)	712.4 (986.6)	0.602	0.602	0.258	0.281
Δ VLF (ms^2)	-271.0 (2030.0)	-150.3 (1230.5)	-12.2 (1066.7)	0.184	0.284	0.228	0.281
Baseline LF (ms^2)	315.1 (678.5)	372.2 (568.8)	272.6 (374.8)	0.143	0.284	0.057	0.281
Standing LF (ms^2)	236.2 (434.4)	366.2 (594.2)	264.8 (370.5)	0.546	0.602	0.281	0.281
Δ LF (ms^2)	-78.9 (343.3)	-5.9 (367.6)	-7.8 (221.8)	0.189	0.284	0.187	0.281

Legend

HRV indices expressed as mean (standard deviation). Statistical analyses performed on $\log_{10}$-transformed values. *Q* indicates ANOVA and ANCOVA *P*-values adjusted with the Benjamini-Hochberg procedure with a 5% False Discovery Rate (FDR). Abbreviations: CN, cognitively normal (controls); aMCI, amnestic mild cognitive impairment; naMCI, non-amnestic mild cognitive impairment; TP, total power; VLF, very low frequency power; LF, low frequency power; Δ HRV index, standing HRV index - baseline HRV index.

Supplementary Table S5 Fisher's r to z transformation test comparing Spearman's correlations between HRV indices and visual rating scales on CT versus MRI

	HA [a]			DWML [b]		Fisher's r to z test		
	CT (*n*=82)	MRI (*n*=56)		CT (*n*=90)	MRI (*n*=56)	Comparison		
	Axial	Axial	Coronal	Axial	Axial	I [c]	II [d]	III [e]
Δ LFn (n.u)	-0.331	-0.313	-0.393	0.277	0.313	0.915	0.696	0.827
Δ LF/HF	-0.331	-0.267	-0.340	0.274	0.302	0.701	0.957	0.865

Legend

[a] aMCI group, [b] naMCI group, [c] HA on CT vs HA on MRI (axial), [d] HA on CT vs HA on MRI (coronal), [e] DWML on CT vs DWML on MRI. All correlations adjusted for hypertension, physical activity and trait anxiety. Abbreviations: HA, hippocampal atrophy; DWML, deep white matter lesions; CT, computed tomography; MRI, magnetic resonance imaging; LFn, low frequency power (normalised); n.u, normalised units; LF/HF, ratio of low frequency power (LF) to high frequency power (HF); Δ HRV index, standing HRV index - baseline HRV index.

Supplementary Table S6 Characteristics of aMCI subjects with and without brain magnetic resonance imaging (MRI)

(A) Clinical characteristics

	MRI (*n*= 54)	No MRI (*n* =28)	*P*-value
Age (years)	79.0 (5.2)	80.4 (5.0)	0.226
Gender, female	40 (74.1)	16 (57.1)	0.118
Education (years)	10.0 (4.2)	9.9 (3.8)	0.881
BMI (kg/m^2)	25.5 (4.2)	25.0 (3.7)	0.446
Hypertension	35 (64.8)	22 (78.6)	0.199
SBP (mmHg)	132.4 (14.1)	133.7 (16.1)	0.840
DBP (mmHg)	75.2 (7.8)	74.5 (8.0)	0.687
Heart rate, baseline (beats/min)	65.2 (10.7)	66.0 (9.7)	0.732
Respiratory rate, baseline (cycles/min)	15.1 (3.5)	15.5 (2.4)	0.316
Respiratory rate, standing (cycles/min)	15.9 (3.4)	16.6 (2.6)	0.316
Smoking	5 (9.3)	1 (3.6)	0.659
Alcohol (AU/day)	1.0 (1.4)	1.0 (1.5)	0.978
Coffee (cups/day)	1.2 (1.1)	1.2 (1.0)	0.739
Physical activity (MET-hrs/week)	53.6 (36.1)	50.0 (35.3)	0.674
Glucose (mg/dl)	92.3 (12.1)	90.5 (11.2)	0.534
Total cholesterol (mg/dl)	215.0 (40.5)	215.7 (39.2)	0.939
LDL cholesterol (mg/dl)	131.1 (36.3)	132.5 (33.0)	0.863
HDL cholesterol (mg/dl)	63.2 (16.2)	61.0 (15.6)	0.395
Triglycerides (mg/dl)	107.8 (44.7)	114.3 (38.9)	0.304
FCVDRP score	16.1 (3.6)	16.8 (2.8)	0.382
FSRP score	13.9 (3.5)	15.1 (4.1)	0.198
Number of medications	4.1 (2.6)	4.7 (2.5)	0.329
Antihypertensive medications			
ACE-I/ARB	27 (50.0)	19 (67.9)	0.122
CCB (dihydropyridines)	11 (20.4)	5 (17.9)	0.785
Diuretics	12 (22.2)	11 (39.3)	0.103
Psychotropic medications			
SSRI	13 (24.1)	6 (21.4)	0.788
Benzodiazepines	10 (18.5)	2 (7.1)	0.205
BADL score	5.5 (0.7)	5.6 (0.5)	0.306
IADL score	6.4 (1.6)	6.3 (1.4)	0.702
MMSE score	25.5 (2.3)	26.4 (1.9)	0.091
MoCA score	21.0 (2.9)	21.3 (2.8)	0.657
CIRS-m score	1.9 (1.1)	2.3 (1.0)	0.096
STPI-T score	17.9 (5.2)	18.4 (4.6)	0.492
GDS-s score	3.0 (2.7)	3.5 (3.0)	0.488
VAS stress score	28.6 (22.9)	20.9 (19.9)	0.157

(B) Peripheral vascular burden and ApoE genotypes

	MRI (*n*=54)	No MRI (*n*=28)	*P*-value
Echocardiography			
LVMI (g/m^2)	86.6 (21.5)	94.6 (17.4)	0.091
LVH %	11 (20.4)	8 (28.6)	0.404
Carotid ultasound			
IMT (mm)	1.0 (0.2)	1.0 (0.1)	0.495
IMT > 0.9 mm	41 (75.9)	25 (89.3)	0.148
Plaque (%)	21.4 (15.5)	20.2 (14.4)	0.775
ApoE Genotype			0.623
E2/E3	2 (3.7)	1 (3.6)	
E3/E3	32 (59.3)	15 (53.6)	
E3/E4	17 (31.5)	12 (42.9)	
E4/E4	3 (5.6)	0 (0)	
Carrier	20 (37.0)	12 (42.9)	0.608

(C) Cerebrovascular burden and hippocampal atrophy on computed tomography

	MRI (*n*=54)	No MRI (*n*=28)	*P*-value
Fazekas' scale score			
PVWML	1.6 (0.8)	1.8 (0.7)	0.187
DWML	1.3 (0.6)	1.5 (0.6)	0.305
Kim's scale score			
Right HA	2.5 (1.0)	2.4 (0.9)	0.740
Left HA	2.4 (1.0)	2.6 (0.9)	0.280
Mean HA	2.4 (1.0)	2.5 (0.9)	0.819

Supplementary Table S7 Characteristics of naMCI subjects with and without brain magnetic resonance imaging (MRI)

(A) Clinical characteristics

	MRI (*n*= 56)	No MRI (*n* = 37)	*P*-value
Age (years)	78.2 (5.3)	79.9 (5.8)	0.153
Gender, female	39 (69.6)	24 (64.9)	0.629
Education (years)	10.3 (5.1)	10.2 (4.8)	0.961
BMI (kg/m^2)	24.7 (4.2)	25.9 (3.4)	0.117
Hypertension	44 (78.6)	24 (64.9)	0.144
SBP (mmHg)	133.0 (14.5)	129.0 (16.0)	0.322
DBP (mmHg)	76.6 (6.9)	74.5 (9.4)	0.214
Heart rate, baseline (beats/min)	67.1 (10.2)	67.7 (9.4)	0.800
Respiratory rate, baseline (cycles/min)	14.9 (2.2)	15.8 (2.7)	0.075
Respiratory rate, standing (cycles/min)	16.3 (2.6)	16.9 (3.0)	0.339
Smoking	4 (7.1)	2 (5.4)	1
Alcohol (AU/day)	1.3 (1.4)	1.2 (1.4)	0.566
Coffee (cups/day)	1.6 (1.1)	1.5 (1.1)	0.771
Physical activity (MET-hrs/week)	80.2 (49.9)	64.9 (36.5)	0.189
Glucose (mg/dl)	92.8 (9.7)	94.1 (11.0)	0.771
Total cholesterol (mg/dl)	220.6 (36.8)	219.4 (31.4)	0.864
LDL cholesterol (mg/dl)	130.9 (29.4)	133.9 (25.4)	0.608
HDL cholesterol (mg/dl)	66.1 (17.0)	62.4 (18.2)	0.194
Triglycerides (mg/dl)	110.7 (48.7)	103.3 (34.8)	0.567
FCVDRP score	16.5 (2.9)	16.0 (3.6)	0.460
FSRP score	14.8 (4.6)	14.0 (4.7)	0.437
Number of medications	4.9 (2.7)	4.2 (2.4)	0.216
Antihypertensive medications			
ACE-I/ARB	36 (64.3)	22 (59.5)	0.638
CCB (dihydropyridines)	13 (23.2)	6 (16.2)	0.413
Diuretics	11 (19.6)	8 (21.6)	0.817
Psychotropic medications			
SSRI	20 (35.7)	10 (27.0)	0.380
Benzodiazepines	15 (26.8)	7 (18.9)	0.382
BADL score	5.4 (0.5)	5.3 (0.5)	0.390
IADL score	6.8 (1.3)	6.7 (1.6)	0.889
MMSE score	27.2 (1.7)	27.8 (2.0)	0.032
MoCA score	22.8 (2.0)	23.0 (1.9)	0.533
CIRS-m score	2.1 (1.2)	2.1 (1.2)	0.696
STPI-T score	20.9 (5.8)	19.1 (5.0)	0.095
GDS-s score	4.1 (2.8)	3.3 (2.9)	0.107
VAS stress score	33.5 (22.4)	24.0 (22.5)	0.039

(B) Peripheral vascular burden and ApoE genotypes

	MRI (*n*=56)	No MRI (*n*=37)	*P*-value
Echocardiography			
LVMI (g/m^2)	101.6 (38.4)	91.6 (21.0)	0.193
LVH %	21 (37.5)	11 (29.7)	0.440
Carotid ultasound			
IMT (mm)	1.1 (0.2)	1.1 (0.2)	0.436
IMT > 0.9 mm	50 (89.3)	32 (86.5)	0.749
Plaque (%)	30.6 (11.7)	28.1 (13.9)	0.372
ApoE Genotype			0.612
E2/E3	4 (7.1)	2 (5.4)	
E3/E3	43 (76.8)	26 (70.3)	
E3/E4	8 (14.3)	9 (24.3)	
E4/E4	1 (1.8)	0 (0)	
Carrier	9 (16.1)	9 (24.3)	0.324

(C) Cerebrovascular burden and hippocampal atrophy on computed tomography

	MRI (*n*=56)	No MRI (*n*=37)	*P*-value
Fazekas' scale score			
PVWML	1.4 (0.6)	1.3 (0.5)	0.687
DWML	1.8 (0.7)	1.9 (0.5)	0.675
Kim's scale score			
Right HA	1.4 (0.7)	1.4 (0.6)	1
Left HA	1.6 (0.7)	1.6 (0.6)	0.708
Mean HA	1.5 (0.6)	1.5 (0.5)	0.780

Supplementary Table S8 Characteristics of cognitively normal subjects in the two enrollments

(A) Clinical characteristics

	Previous (*n*=40)	Current (*n*=38)	*P*-value
Age (years)	77.8 (4.5)	79.0 (5.0)	0.269
Gender, female	33 (82.5)	30 (78.9)	0.691
Education (years)	11.4 (4.4)	11.5 (4.5)	0.976
BMI (kg/m^2)	24.1 (3.0)	24.3 (2.9)	0.713
Hypertension	21 (52.5)	20 (52.6)	0.991
SBP (mmHg)	134.8 (19.2)	132.9 (12.2)	0.607
DBP (mmHg)	75.2 (8.7)	76.5 (7.7)	0.489
Heart rate, baseline (beats/min)	65.2 (8.7)	65.0 (9.4)	0.737
Respiratory rate, baseline (cycles/min)	14.0 (2.4)	15.0 (2.6)	0.111
Respiratory rate, standing (cycles/min)	14.9 (2.7)	16.2 (3.0)	0.048
Smoking	2 (5.0)	5 (13.2)	0.257
Alcohol (AU/day)	1.3 (1.6)	1.3 (1.4)	0.876
Coffee (cups/day)	1.5 (1.0)	1.7 (1.3)	0.756
Physical activity (MET-hrs/week)	68.4 (39.3)	67.9 (34.6)	0.956
Glucose (mg/dl)	87.7 (9.8)	93.8 (13.5)	0.109
Total cholesterol (mg/dl)	222.9 (37.8)	215.8 (37.0)	0.422
LDL cholesterol (mg/dl)	137.6 (33.0)	130.1 (29.0)	0.311
HDL cholesterol (mg/dl)	69.2 (20.5)	65.6 (17.9)	0.463
Triglycerides (mg/dl)	104.0 (32.4)	111.2 (50.8)	0.973
FCVDRP score	15.6 (3.4)	15.9 (2.9)	0.708
FSRP score	13.5 (4.2)	13.9 (4.3)	0.642
Number of medications	3.3 (1.7)	3.5 (2.0)	0.631
Antihypertensive medications			
ACE-I/ARB	16 (40.0)	15 (39.5)	0.962
CCB (dihydropyridines)	3 (7.5)	6 (15.8)	0.305
Diuretics	7 (17.5)	7 (18.4)	0.916
Psychotropic medications			
SSRI	9 (22.5)	6 (15.8)	0.452
Benzodiazepines	8 (20.0)	5 (13.2)	0.418
BADL score	5.5 (0.5)	5.4 (0.5)	0.798
IADL score	7.4 (1.2)	7.5 (1.1)	0.830
MMSE score	28.6 (1.0)	28.6 (1.0)	0.737
MoCA score	26.5 (1.9)	24.6 (0.7)	<0.001
CIRS-m score	2.5 (1.3)	2.0 (1.1)	0.129
STPI-T score	19.5 (5.7)	17.4 (5.6)	0.077
GDS-s score	3.4 (3.1)	3.1 (2.9)	0.512
VAS stress score	33.7 (22.4)	31.2 (22.1)	0.581

(B) Peripheral vascular burden and ApoE genotypes

	Previous (*n*=40) †	Current (*n*=38) ††	*P*-value
Echocardiography			
LVMI (g/m^2)	84.0 (17.5)	82.6 (13.8)	0.964
LVH %	8 (20.0)	7 (18.4)	0.860
Carotid ultasound			
IMT (mm)	1.0 (0.1)	1.0 (0.1)	0.584
IMT > 0.9 mm	35 (87.5)	33 (86.8)	1
Plaque (%)	22.0 (15.5)	22.4 (13.7)	0.774
ApoE Genotype			0.722
E2/E3	4 (12.5)	1 (4.3)	
E3/E3	23 (71.9)	19 (82.6)	
E3/E4	5 (15.6)	3 (13.0)	
E4/E4	0 (0)	0 (0)	
Carrier	5 (15.6)	3 (13.0)	1

† ApoE genotyping available for *n*=32, †† ApoE genotyping available for *n*=23.

(C) Cerebrovascular burden and hippocampal atrophy on computed tomography (CT)

	Previous (*n*=40)†	Current (*n*=38)† †	*P*-value
Fazekas' scale score			
PVWML	1.4 (0.5)	1.2 (0.4)	0.463
DWML	1.3 (0.6)	1.2 (0.4)	0.851
Kim's scale score			
Right HA	1.3 (0.6)	1.6 (1.0)	0.528
Left HA	1.5 (0.5)	1.9 (0.9)	0.175
Mean HA	1.4 (0.5)	1.7 (0.9)	0.251

† CT scans available for *n*=16, † † CT scans available for *n*=18.

E) Neuropsychological tests Z-scores

	Previous (*n*=40)	Current (*n*=38)	*P*-value
Prose-delayed recall	0.70 (0.92)	0.60 (0.78)	0.708
ROCF-delayed recall	0.02 (0.79)	-0.10 (0.81)	0.494
Bell Test	-0.28 (1.23)	-0.35 (1.03)	0.452
Digit Cancellation test	0.42 (0.55)	0.51 (0.55)	0.461
Executive function †	0.02 (0.42)	0.02 (0.33)	0.935
Language †	0.17 (0.48)	0.36 (0.60)	0.137
Visuospatial skills †	0.51 (0.28)	0.58 (0.33)	0.143
Ideomotor praxis †	0.79 (0.37)	0.90 (0.25)	0.277

† Composite score.

Legend to Supplementary Tables S6, S7 and S8

Continuous variables expressed as mean (standard deviation), categorical variables expressed as n (%). Student's t-test or Mann-Whitney's test for continuous variables. Chi-squared or Fisher's exact test for categorical variables. Abbreviations: BMI, body mass index; SBP, systolic blood pressure; DBP, diastolic blood pressure; AU, alcohol units (1 AU=10 g of alcohol); MET, metabolic equivalent (energy expenditure index, 1 MET= 1 $kcal \cdot kg^{-1} \cdot h^{-1}$; LDL, low density lipoprotein; HDL, high density lipoprotein; FCVDRP, Framingham cardiovascular disease risk profile (score range -6-38, higher scores indicate higher risk); FSRP, Framingham stroke risk profile (score range 0-48 , higher scores indicate higher risk); ACE-I, angiotensin converting enzyme inhibitors; ARB, angiotensin II receptor blockers; CCB, calcium channel blockers; SSRI, selective serotonin reuptake inhibitors; BADL, basic activities of daily living (score range 0-6, higher scores indicate greater functional independence); IADL, instrumental activities of daily living (score range 0-8, higher scores indicate greater functional independence); MMSE, mini mental state examination (score range 0-30, higher scores indicate better cognitive function); MoCA, Montreal cognitive assessment (score range 0-30, higher scores indicate better cognitive function); CIRS-m, cumulative illness rating scale morbidity (score range 0-13, higher scores indicate more severe comorbidity); STPI-T, state trait personality inventory-trait anxiety subscale (score range 10-40, higher scores indicate greater trait anxiety); GDS-s, geriatric depression scale short form (score range 0-15, higher scores indicate greater depressive symptoms); LVMI, left ventricular mass index; LVH, left ventricular hypertrophy; IMT, intima-media thickness; PVWML, periventricular white matter lesions (score range 0-3, higher scores indicate greater WML load); DWML, deep white matter lesions (score range 0-3, higher scores indicate greater WML load); HA, hippocampal atrophy (score range 0-4, higher scores indicate greater atrophy); FI, frontoinsula; IA, insular atrophy (score range 0-3, higher scores indicate greater atrophy); carrier, carrying at least one E4 allele; ROCF, Rey-Osterrieth complex figure.

Supplementary Table S9 Reasons for neuroimaging in cognitively normal subjects

	CT (*n*=34)	MRI (*n*=14)
Falls	8 (23.5)	0 (0)
Postural instability	0 (0)	2 (14.3)
Subjective cognitive complaints	8 (23.5)	2 (14.3)
Cephalea	9 (26.5)	5 (35.7)
Tinnitus	3 (8.8)	2 (14.3)
Vertigo	2 (5.9)	1 (7.1)
Paresthesias	2 (5.9)	1 (7.1)
Suspected transient ischaemic attack	2 (5.9)	1 (7.1)

Legend

Data expressed as n (%). Abbreviations: CT, computed tomography; MRI, magnetic resonance imaging.

Supplementary Table S10 Neuropsychological test battery

Cognitive domain	Neuropsychological test	Reference
Attention	Bell Test<br>Digit Cancellation Test	Vallar, G., et al. (1994)[214]<br>Spinnler, H. & Tognoni, G. (1987)[215]
Episodic memory	Prose-delayed recall<br>ROCF-delayed recall	Carlesimo, G. A., et al. (2002)[216]
Executive functions	Digit Span Forwards<br>Digit Span Backwards	Orsini, A., et al. (1987)[217]<br>Monaco, M., et al. (2013)[218]
	Trail-Making Test A<br>Trail-Making Test B	Giovagnoli, A. R., et al. (1996)[219]
	Weigl's Test	Spinnler, H. & Tognoni, G. (1987)[215]
	Cognitive Estimates-total<br>Cognitive Estimates-bizarre	Della Sala, S., et al. (2003)[220]
	Raven's coloured matrices	Spinnler, H.& Tognoni, G. (1987)[215]
	Letter fluency	Novelli, G., et al. (1986)[221]
Language	Category fluency<br>Picture naming<br>Token Test	Spinnler, H. & Tognoni, G. (1987)[215]<br>Laiacona, M., et al. (1993)[222]<br>Spinnler, H. & Tognoni, G. (1987)[215]
Visuospatial skills	ROCF-copy<br>Copy of geometric figures	Caffarra, P., et al. (2002)[223]<br>Spinnler, H. &Tognoni, G. (1987)[215]
Ideomotor praxis	De Renzi's Test - right upper limb<br>De Renzi's Test - left upper limb	De Renzi, E., et al. (1980)[224]

Legend

ROCF, Rey-Osterrieth complex figure.

Supplementary Table S11 Neuropsychological test scores across cognitive domains in the study groups

	CN (*n*=78)	aMCI (*n*=82)	naMCI (*n*=93)	*P*-value
Attention				
Bell Test	34.3 (0.8)	32.0 (3.7)	32.0 (3.3)	<0.001 [b***,c***]
Digit Cancellation Test	54.0 (5.1)	49.6 (6.1)	47.6 (6.9)	<0.001 [b***,c***]
Episodic memory				
Prose-delayed recall	7.4 (1.3)	2.8 (2.3)	6.9 (1.2)	<0.001 [a***,b***]
ROCF-delayed recall	22.6 (5.6)	8.8 (6.5)	18.0 (5.3)	<0.001 [a***,b, ***c***]
Executive functions				
Digit Span Forwards	5.8 (0.9)	5.1 (1.0)	5.2 (0.9)	<0.001 [b***,c***]
Digit Span Backwards	4.5 (0.6)	3.9 (1.0)	3.7 (0.8)	<0.001 [b***,c***]
Trail-Making Test A	28.0 (12.7)	48.1 (30.2)	41.0 (24.2)	<0.001 [b,***c***]
Trail-Making Test B	57.2 (34.0)	203.0 (123.4)	171.8 (126.1)	<0.001 [b***,c***]
Weigl's Test	12.0 (1.8)	8.8 (2.9)	9.2 (2.7)	<0.001 [b***,c***]
Cognitive Estimates-total	11.1 (1.9)	16.0 (3.5)	16.1 (3.1)	<0.001 [b***,c***]
Cognitive Estimates-bizarre	1.8 (1.0)	3.9 (2.0)	4.3 (1.8)	<0.001 [b***,c***]
Raven's coloured matrices	33.4 (3.8)	27.0 (5.5)	27.4 (5.8)	<0.001 [b***,c***]
Letter fluency	35.9 (8.1)	27.7 (9.0)	29.3 (9.3)	<0.001 [b***,c***]
Language				
Category fluency	19.4 (3.9)	14.1 (4.3)	16.2 (4.3)	<0.001 [a**,b***,c***]
Picture naming	75.1 (2.9)	68.3 (7.9)	69.3 (7.3)	<0.001 [b***,c***]
Token Test	33.9 (1.4)	30.7 (2.4)	30.9 (2.4)	<0.001 [b***,c***]
Visuospatial skills				
ROCF-copy	35.6 (1.4)	30.9 (6.7)	31.0 (5.7)	<0.001 [b***,c***]
Copy of geometric figures	13.7 (0.5)	12.4 (1.6)	12.4 (1.7)	<0.001 [b***,c***]
Ideomotor praxis				
De Renzi's Test - right upper limb	71.7 (0.7)	70.0 (3.2)	70.6 (1.9)	<0.001 [b***,c***]
De Renzi's Test - left upper limb	71.2 (1.5)	69.8 (3.5)	70.3 (2.2)	<0.001 [b***,c***]

Legend

Neuropsychological test scores (demographically-adjusted) expressed as mean (standard deviation). Higher scores indicate better cognitive performance except for Trail-Making and Cognitive Estimates tests for which higher scores indicate worse cognitive performance. ANOVA or Kruskall-Wallis test with Bonferroni-corrected pairwise comparisons. [a] significant difference between aMCI and naMCI, [b] significant difference between aMCI and CN, [c] significant difference between naMCI and CN.*** $p \leq 0.001$, ** $p < 0.01$. Abbreviations: CN, cognitively normal (controls); aMCI, amnestic mild cognitive impairment; naMCI, non-amnestic mild cognitive impairment; ROCF, Rey-Osterrieth complex figure.

Supplementary Table S12 Categorisation of MCI subjects according to number and type of cognitive domains impaired

	aMCI (*n*=82)		naMCI (*n*=93)	
	SD	MD	SD	MD
Subjects [a]	13 (15.9)	69 (84.1)	30 (32.3)	63 (67.7)
Non-memory domain impaired [b]				
Attention	-	30 (43.5)	3 (10)	36 (57.1)
Executive function	-	62 (89.9)	25 (83.3)	60 (95.2)
Language	-	44 (63.8)	1 (3.3)	34 (54.0)
Visuospatial skills	-	29 (42.0)	1 (3.3)	35 (55.6)
Ideomotor praxis	-	1 (1.4)	0 (0)	1 (1.6)

Legend

[a] Expressed as number (%) where % is relative to the main MCI subtype (aMCI or naMCI),

[b] Expressed as number (%) where % is relative to the MCI MD or SD subtype. Percentages do not add up to 100 since impairments in cognitive domains are not mutually exclusive. Abbreviations: aMCI, amnestic mild cognitive impairment; naMCI; non-amnestic mild cognitive impairment; SD, single domain; MD, multiple domain.
